# Cost and cost-effectiveness of a universal HIV testing and treatment intervention in Zambia and South Africa: evidence and projections from the HPTN 071 (PopART) trial

**DOI:** 10.1016/S2214-109X(21)00034-6

**Published:** 2021-03-12

**Authors:** Ranjeeta Thomas, William J M Probert, Rafael Sauter, Lawrence Mwenge, Surya Singh, Sarah Kanema, Nosivuyile Vanqa, Abigail Harper, Ronelle Burger, Anne Cori, Michael Pickles, Nomtha Bell-Mandla, Blia Yang, Justin Bwalya, Mwelwa Phiri, Kwame Shanaube, Sian Floyd, Deborah Donnell, Peter Bock, Helen Ayles, Sarah Fidler, Richard J Hayes, Christophe Fraser, Katharina Hauck, HPTN 071 (PopART) study team

**Affiliations:** a Department of Health Policy, London School of Economics and Political Science, London, UK; b Big Data Institute, Li Ka Shing Centre for Health Information and Discovery, Nuffield Department of Medicine, University of Oxford, Oxford, UK; c Zambart, University of Zambia, Lusaka, Zambia; d Health Economics Research Centre, Nuffield Department of Population Health, University of Oxford, Oxford, UK; e Desmond Tutu Tuberculosis Centre, Department of Paediatrics and Child Health, Stellenbosch University, Cape Town, South Africa; f Department of Economics, Stellenbosch University, Cape Town, South Africa; g Medical Research Council Centre for Global Infectious Disease Analysis, School of Public Health, Imperial College London, London, UK; h Abdul Latif Jameel Institute for Disease and Emergency Analytics, School of Public Health, Imperial College London, London, UK; i Department of Infectious Disease, Imperial College London, London, UK; j Department of Infectious Disease Epidemiology, Faculty of Epidemiology and Population Health, London School of Hygiene & Tropical Medicine, London, UK; k Department of Clinical Research, Faculty of Infectious and Tropical Diseases, London School of Hygiene & Tropical Medicine, London, UK; l Fred Hutchinson Cancer Research Center, Seattle, WA, USA

## Abstract

**Background:**

The HPTN 071 (PopART) trial showed that a combination HIV prevention package including universal HIV testing and treatment (UTT) reduced population-level incidence of HIV compared with standard care. However, evidence is scarce on the costs and cost-effectiveness of such an intervention.

**Methods:**

Using an individual-based model, we simulated the PopART intervention and standard care with antiretroviral therapy (ART) provided according to national guidelines for the 21 trial communities in Zambia and South Africa (for all individuals aged >14 years), with model parameters and primary cost data collected during the PopART trial and from published sources. Two intervention scenarios were modelled: annual rounds of PopART from 2014 to 2030 (PopART 2014–30; as the UNAIDS Fast-Track target year) and three rounds of PopART throughout the trial intervention period (PopART 2014–17). For each country, we calculated incremental cost-effectiveness ratios (ICERs) as the cost per disability-adjusted life-year (DALY) and cost per HIV infection averted. Cost-effectiveness acceptability curves were used to indicate the probability of PopART being cost-effective compared with standard care at different thresholds of cost per DALY averted. We also assessed budget impact by projecting undiscounted costs of the intervention compared with standard care up to 2030.

**Findings:**

During 2014–17, the mean cost per person per year of delivering home-based HIV counselling and testing, linkage to care, promotion of ART adherence, and voluntary medical male circumcision via community HIV care providers for the simulated population was US$6·53 (SD 0·29) in Zambia and US$7·93 (0·16) in South Africa. In the PopART 2014–30 scenario, median ICERs for PopART delivered annually until 2030 were $2111 (95% credible interval [CrI] 1827–2462) per HIV infection averted in Zambia and $3248 (2472–3963) per HIV infection averted in South Africa; and $593 (95% CrI 526–674) per DALY averted in Zambia and $645 (538–757) per DALY averted in South Africa. In the PopART 2014–17 scenario, PopART averted one infection at a cost of $1318 (1098–1591) in Zambia and $2236 (1601–2916) in South Africa, and averted one DALY at $258 (225–298) in Zambia and $326 (266–391) in South Africa, when outcomes were projected until 2030. The intervention had almost 100% probability of being cost-effective at thresholds greater than $700 per DALY averted in Zambia, and greater than $800 per DALY averted in South Africa, in the PopART 2014–30 scenario. Incremental programme costs for annual rounds until 2030 were $46·12 million (for a mean of 341 323 people) in Zambia and $30·24 million (for a mean of 165 852 people) in South Africa.

**Interpretation:**

Combination prevention with universal home-based testing can be delivered at low annual cost per person but accumulates to a considerable amount when scaled for a growing population. Combination prevention including UTT is cost-effective at thresholds greater than $800 per DALY averted and can be an efficient strategy to reduce HIV incidence in high-prevalence settings.

**Funding:**

US National Institutes of Health, President's Emergency Plan for AIDS Relief, International Initiative for Impact Evaluation, Bill & Melinda Gates Foundation.

## Introduction

In 2018, approximately 38 million people were living with HIV infection worldwide, with 1·7 million new infections that year.^1^ HIV incidence is decreasing worldwide, but is unlikely to reach the UNAIDS Fast-Track target of less than 200 000 new infections in 2030.^2^ Steep reductions in incidence are needed to curb the HIV epidemic and its associated financial, societal, and health costs. A universal HIV testing and treatment (UTT) strategy that includes home-based counselling and testing (HBCT) and linkage to care in high-prevalence communities has been proposed as an important component of HIV prevention programmes.^3^, ^4^ Four randomised population-based trials in sub-Saharan Africa analysed the effectiveness of UTT. Two of these trials (Treatment as Prevention^5^ and Sustainable East Africa Research in Community Health^6^) showed no effect on HIV incidence; whereas another of the trials (Botswana Combination Prevention Project)^7^ showed a 31% decrease in HIV incidence after 3 years in intervention communities compared with control communities (n=15 per group). The fourth trial, the HIV Prevention Trials Network (HPTN) 071 (PopART) study, evaluated whether a combination prevention strategy including universal testing via HBCT and antiretroviral therapy (ART) could be effectively implemented at a population level in Zambia and South Africa.^8^ Within 2 years, in adults aged 18–44 years, HIV incidence was reduced by around 20% in communities receiving the combination prevention package (n=14), compared with communities receiving standard care (n=7).^8^

**Research in context**

**Evidence before this study**

We searched PubMed and Embase on Nov 20, 2019, for health economic analyses of home-based HIV counselling and testing (HBCT) and linkage to care published between Jan 1, 2000, and Sept 13, 2019, with the terms “HIV” AND “Africa South of the Sahara” AND (“home” OR “community” OR “mass screening” OR “testing” OR “screen” OR “diagnosis” OR “counselling”) AND (“cost” OR “cost-effectiveness” OR “cost-utility” OR “cost-benefit”). We included studies that assessed HBCT against facility-based HIV prevention and care, and excluded studies on mobile testing, partner notification, and index-linked testing of partners or children of HIV-infected individuals. We excluded studies that compared universal HIV testing and treatment (UTT) against standard care with antiretroviral therapy (ART) conditional on CD4 eligibility criteria. These studies would not be comparable with our study incorporating universal provision of ART as standard care for most years of the projection horizon. We included studies that assessed HBCT on its own or in combination with screening for an HIV co-infection including tuberculosis, but not those that combined it with other interventions such as HIV self-testing or diagnosis for other conditions. We included modelling and simulation studies that were targeted at the general population, that considered effect on treatment outcomes of HIV-infected individuals and on the incidence of new HIV infections, and that had documented the methodology, cost estimates, frequency of testing rounds, and projection horizon. We included studies that measured effectiveness in terms of disability-adjusted life-years (DALYs) averted or quality-adjusted life-years (QALYs) gained, life-years gained, or infections averted, but excluded studies that measured effectiveness in terms of intervention uptake or HIV-positive cases identified. Five studies in Uganda and South Africa fulfilled our inclusion criteria. In modelling projections of various combination interventions, estimates of cost-effectiveness varied widely, at US$860–1710 per QALY gained or DALY averted, $8639–22 000 per infection averted, $474–3400 per life-year saved, and cost-effectiveness thresholds greater than $1690 per DALY averted.

**Added value of this study**

Limited and conflicting evidence is available on the cost-effectiveness of combination prevention interventions including UTT. The findings of this study provide important evidence on the benefits and costs of UTT in high-prevalence communities. Existing modelling studies are problematic to compare because of differences across studies, most notably the country settings, features of the intervention that were modelled, target population, baseline prevalence, frequency of HBCT, and model projection horizon. Previous studies also had to rely heavily on simulations and secondary data sources. Until now, to the best of our knowledge, there has been no evidence from large-scale population randomised studies, and no large-scale evidence on the effectiveness of a combination prevention intervention in reducing HIV incidence. This study used primary data collected as part of the HPTN 071 (PopART) trial of a combination prevention intervention including UTT, delivered via a home-based HIV testing approach in Zambia and South Africa. We have evaluated the cost-effectiveness of the actual trial intervention implemented between 2014 and 2017, projecting outcomes until 2030. In addition, we modelled the cost-effectiveness of an annual campaign that is sustained for 17 years between 2014 and 2030. We projected that the PopART intervention implemented annually up to 2030 has almost 100% probability of being cost-effective at cost-effectiveness thresholds greater than $800 per DALY averted in Zambia and South Africa.

**Implications of all the available evidence**

The estimates of cost-effectiveness from this study are more economically favourable than those of previous studies, probably due to the projected reductions in HIV incidence caused by the intervention, reduction in costs of ART in the past years, and the scale at which PopART was implemented. The intervention was not cost-saving in either scenario, explained by repeated rounds of HBCT, sustained high expenditures on treatment from improved linkage to care, and the near normal life expectancy of HIV-infected individuals receiving ART. The affordability of PopART is an important consideration. Although the estimated unit costs of the intervention per person covered are low, they accumulate to a considerable amount when projected for the total population (age >14 years) to be covered. The optimal frequency of intervention rounds is an important consideration. Our findings show that three annual rounds cannot sustain the initial reduction in incidence, and numbers of new infections approach those under standard care after 13 years. Previous evidence has shown that prevention interventions that prioritise specific subpopulations are often more cost-effective than interventions that target the general population. Our results show that a population-level combination prevention strategy can be economically efficient.

To date, PopART is the largest population-level randomised controlled trial of a combination prevention intervention against HIV that resulted in reduced incidence. However, little evidence is available on the costs, cost-effectiveness, and budgetary implications of such an intervention. An estimate for the cost per person tested via HBCT is US$22·8 (SD $14·5), averaged across 14 studies, with an estimated minimum cost of $6 and maximum cost of $55.^9^ Evidence on cost-effectiveness of HBCT is also limited and comes from small studies with varying estimates.^10^, ^11^, ^12^, ^13^, ^14^ Estimates of incremental cost-effectiveness ratios (ICERs) from three modelling studies were $860–1710 per disability-adjusted life-year (DALY) averted (or quality-adjusted life-year gained [QALY]),^11^, ^13^ and $8639–22 000 per infection averted.^11^, ^14^ In this Article, our aim was to provide evidence from the PopART intervention on the costs, cost-effectiveness, and budget implications of combination HIV prevention including UTT.

## Methods

### Study design

The HPTN 071 (PopART) trial took place between 2013 and 2018 (intevention period from November, 2013, to December, 2017), in 21 large urban communities in Zambia (n=12) and South Africa (n=9) (total population approximately 1 million). The 21 trial communities were grouped in seven matched triplets based on location and HIV prevalence. Within each triplet communities were randomly allocated to one of three trial arms: two intervention arms (A and B) and a control arm (C). The combination prevention intervention included, amongst other components, HBCT delivered by community HIV care provider (CHiP) teams, who also supported linkage to HIV care, promoted ART adherence, provided condoms, and promoted a package of prevention strategies among HIV-negative individuals, including voluntary medical male circumcision (VMMC). In addition, one intervention arm of the trial (seven communities in arm A) provided ART irrespective of CD4 count threshold, while the other intervention arm (seven communities in arm B) provided ART according to national guidelines (CD4 threshold of 350 cells per μL in 2013, which increased to 500 cells per μL in 2014). Due to the change in national guidelines to universal ART, the two intervention arms were equivalent from April, 2016 onward in Zambia and October, 2016 onward in South Africa. The trial has been described in detail previously.^8^, ^15^ Ethical approval for the trial was granted by ethics committees at the London School of Hygiene & Tropical Medicine (London, UK), the University of Zambia (Lusaka, Zambia), and Stellenbosch University (Stellenbosch, South Africa).

For our cost-effectiveness analysis of PopART, we used an individual-based simulation model (PopART-IBM),^16^ specifically developed to model the trial and informed extensively by data collected during the trial. In each intervention community (arms A and B), the model simulates the HIV epidemic, standard care (for HIV counselling and testing and VMMC) and the following components of the PopART intervention: universal HBCT, linkage to care, promotion of ART adherence, and VMMC delivered by CHiPs along with universal ART in arm A and ART according to national guidelines in arm B. Thus, the two modelled intervention arms differ in ART provision for the first 2 years (2014–15) and are equivalent for the remainder of the projection period. We used the model to estimate the combined cost and cost-effectiveness of both intervention arms in PopART compared with a counterfactual of standard care provided at government clinics, with ART offered according to national guidelines.

We modelled two scenarios in men and women (appendix 1 p 10) over a time horizon up to 2030 (as the UNAIDS Fast-Track target year). The first scenario, PopART 2014–30, modelled the PopART intervention implemented in annual rounds from 2014 to 2030, with the counterfactual simulating standard care. The second scenario, PopART 2014–17, modelled the PopART intervention implemented in three annual rounds from 2014 to 2017, over the actual trial period and then discontinued up to 2030, with the counterfactual simulating standard care.

### PopART-IBM

The PopART-IBM (described in appendix 1 [pp 1–5] and a preprint paper^16^) simulates every individual aged older than 14 years in a growing heterosexual population of approximately the same size as each modelled trial community. Demography is modelled from country-specific, age-specific, and sex-specific mortality and fertility rates from the UN Population Division.^17^ Partnership formation and dissolution are parameterised with data from extended questionnaires on sexual behaviour collected during the trial.^8^ Key model and calibration parameters are listed in table 1.

**Table 1:** Key model and calibration parameters in the PopART individual-based simulation model

	**Value or range explored**	**Notes**
**HIV-related parameters**		
Start of HIV epidemic, year	1975 (Zambia), 1980 (South Africa)	..
Average annual hazard of an (uncircumcised) man becoming HIV-positive from an HIV-positive partner who has maximal set-point viral load	0·05–0·30	Hollingsworth et al (2008);^18^ Fraser at al (2007)^19^
Relative infectivity by HIV stage (relative to CD4 count of ≥500 cells per μL)	1·00 (CD4 350–500), 1·00 (CD4 200–350), 2·34 (CD4 <200), 5·30 (AEHI)	Bellan et al (2015)^20^
Duration of AEHI, years	0·08–0·25	Bellan et al (2015)^20^
Relative infectivity of male-to-female transmission (compared with female-to-male)	1·0–3·0	Boily et al (2009)^21^
**HIV care-related parameters**		
Probability of a women having an HIV test under standard care in 2000–06	0·1–0·2	Estimated in the calibration; probability is for a period of 6 years
Annual probability of a women having an HIV test from 2006 onwards under standard care	0·05–0·40	Estimated in the calibration
Relative probability of a man having an HIV test under standard care (at any time; compared with women)	0·4–1·1	Estimated in the calibration
Probability of collecting HIV test results from an HIV test under standard care	0·97–1·00	Demographic and Health Survey 2013 (Zambia)
Probability of collecting a CD4 test result under standard care	0·75–0·95	Lower limit is from Mugglin et al (2012);^22^ higher limit assumed
Mean time to starting ART after an HIV-positive test delivered under standard care (conditional on starting ART), years	0·4–0·7	Estimated in the calibration
Probability of a women staying virally suppressed for life after ART initiation	0·65–0·90	Estimated in the calibration
Relative probability of a man staying virally suppressed for life (compared with a woman)	0·6–1·0	Estimated in the calibration
After ART initiation, probability of an individual becoming virally unsuppressed due to suboptimal ART adherence	0·1	Vinikoor et al (2014)^23^
Relative infectivity of an individual on ART (compared with not being on ART)	0·5 (early ART),* 0·7 (virally unsuppressed), 0·0 (virally suppressed)	Values assumed; no transmission from individuals who are virally suppressed
Probability of a man accepting VMMC after an HIV-negative test result†	0·4	Assumption cross-checked against population cohort† data
Reduction in susceptibility to HIV infection for a circumcised male	0·6 (VMMC), 0·0 (traditional male circumcision)	Population cohort‡ data
**Partnership-related parameters**		
Risk assortativity	0·05–0·95	The propensity for individuals within the same risk group§ to form partnerships with those in the same risk group; estimated in the calibration
Relative number of sexual partners (compared with self-report)	0·625–5·000	Estimated in the calibration; used to account for misreporting of sexual partners

AEHI=acute and early HIV infection. ART=antiretroviral therapy. VMMC=voluntary medical male circumcision.

* The 2-month period after initiating ART when an individual is not fully virally suppressed.

† Assumed to be a fixed probability of VMMC acceptance across the intervention and counterfactual simulations.

‡ A random sample of ∼2500 individuals aged 18–44 years per trial community (n=21), within which the primary endpoint of the trial was measured.

§ Representing level of sexual activity.

HIV is introduced into the simulated population between 1975 and 1980 and HIV transmission is assumed to only occur in serodiscordant couples. HIV disease progression without ART is assumed to occur at rates estimated from the AIDS therapy evaluation in the Netherlands study (appendix 1 pp 3–4).^24^ In intervention communities, CHiP teams are assumed to visit individuals and offer the PopART intervention package with a coverage that matches trial data, stratified by age and sex. HIV testing, with assumed 100% sensitivity and specificity, is done at each CHiP visit, and individuals with an HIV-positive test result are offered ART irrespective of CD4 cell count threshold in arm A communities, while in arm B communities individuals are offered ART according to national guidelines. Time until ART initiation, after an HIV-positive test result as part of a visit from a CHiP, is modelled from trial data. In the counterfactual scenario of standard care, the epidemic without the PopART intervention is simulated with the same parameters (table 1) apart from those affected by PopART (testing coverage, ART eligibility, and dropout rates). Repeat CD4 testing is simulated for those not immediately eligible for ART in both the intervention and standard care communities (before the introduction of universal ART either as part of the PopART intervention from the start of the trial in arm A, or from 2016 in arm B and the counterfactual).

Individuals starting ART can either become virally suppressed, virally unsuppressed, or dropout of care, and the risk of HIV transmission to partners is dependent on an individual's position within the care cascade. VMMC is offered by CHiPs to any HIV-negative male after a negative HIV test (table 1). Thus VMMC uptake in the PopART intervention scenarios can occur after a negative test by CHiPs or at a health facility, making it different from uptake under standard care. VMMC is assumed to offer a 60% reduction in susceptibility,^25^, ^26^ while traditional male circumcision is assumed to offer no protection according to PopART data (appendix 1 p 3). Prevalence of traditional male circumcision differs by community according to trial data.

The PopART-IBM is calibrated separately to each of the 14 trial intervention communities via approximate Bayesian computation.^27^ The calibration approach (appendix 1 pp 4–5) provides 1000 simulations for the intervention and counterfactual standard care in each community. For calibration, the PopART-IBM uses trial data and national historical surveys (table 1 and appendix 1 p 5), all stratified by age and sex.

The predictive ability of the model has previously been compared against primary and selected secondary endpoints of the trial. Projections from PopART-IBM included the estimated primary endpoint of the trial (relative reduction in cumulative incidence between 12 and 36 months of the trial) in arm B communities, both before and after trial unblinding, and the estimated primary endpoint in arm A communities after trial unblinding.^28^ The model calibrated well to the majority of the data over time including age-stratified and sex-stratified HIV prevalence, proportion of people living with HIV aware of their status, proportion of people on ART among those aware of their HIV status, and proportion of people virally suppressed among those living with HIV. Detailed results for an intervention community in Zambia are available in the preprint paper by Pickles and colleagues.^16^

Projected HIV incidence under PopART 2014–30 and PopART 2014–17 is presented by country and sex along with 2·5% and 97·5% quantiles of mean incidence from the PopART-IBM calibration.

### Costs

We did microcosting studies for both countries to derive the costs of the PopART intervention during the trial. We collected costs from a health-care provider perspective using data from study records, expense reports, and consultations with staff at all trial HIV care facilities. A combination of ingredients-based and activity-based costing was used to estimate the cost per person per year covered by CHiPs in the community, the additional costs of individuals accepting the CHiP intervention, and the costs for persons testing HIV-positive or HIV-negative. To isolate the costs of HBCT from other components of the combination prevention package and research activities, a detailed time-and-motion study of CHiP activities was done (appendix 1 pp 5–7).

To estimate the cost of ART per person per year, detailed costing surveys of all HIV clinics (appendix 1 pp 7–8) participating in the trial were implemented in 2015–17. A facility costing tool was developed specifically for this study, and collected data on staff numbers, salary scales, age and size of buildings, laboratory tests done, medical and non-medical equipment, antiretroviral drug dispensing and stock levels, drugs dispensed for the prevention and treatments of opportunistic infections, and general costs for buildings, maintenance, vehicles, and utilities. Data were collected from clinic records, interviews with key personnel at the facility, local and national government offices, non-governmental institutions, and providers of supply chains. Data on patient numbers, drug dispensing, and laboratory tests were collated via review of administrative paper records and electronic monitoring records. Costs of HIV testing, CD4 cell count testing, health-care use (for those initiating ART, those not on ART, and end-of-life care), and VMMC were based on published estimates for Zambia and South Africa (table 2). All costs are expressed in 2017 US$.

**Table 2:** Cost parameters

	**Zambia**				**South Africa**			
	Point estimate	PSA distribution*	Range†	Source	Point estimate	PSA distribution*	Range†	Source
Basic cost per person covered by CHiPs	$5·08	Uniform distribution	−20% to 20%‡	PopART study data	$6·36	Uniform distribution	+ or −20%‡	PopART study data
Cost per person testing HIV-positive by CHiPs	$14·07	Uniform distribution	−20% to 20%‡	PopART study data	$16·91	Uniform distribution	+ or −20%‡	PopART study data
Cost per person testing HIV-negative by CHiPs	$9·08	Uniform distribution	−20% to 20%‡	PopART study data	$10·76	Uniform distribution	+ or −20%‡	PopART study data
Cost per person for HIV counselling and testing at health-care facility	$4·32	Gamma	α=5·50, β=0·80	Mwenge et al (2017)^29^	$4·88	Gamma	α=14·75, β=0·38	Point estimate from PopART study data; range based on Meyer-Rath et al (2019)^30^
Cost per CD4 cell count test	$6·48	Gamma	α=38·07, β=0·16	Cassim et al (2014)^31^	$6·18	Gamma	α=38·07, β=0·16	Point estimate from PopART study data; range based on Cassim et al (2014)^31^
Cost of ART per person per year	$212·50	Gamma	α=4·80, β=44·38	PopART study data	$315·39	Gamma	α=9·01, β=35·02	PopART study data
Cost per voluntary medical male circumcision	$56·16	Gamma	α=25·00, β=2·25	Vandament et al (2016)^32^	$129·07	Gamma	α=179·64, β=0·72	Tchuenche et al (2016)^33^
Cost of health care for HIV-positive person not on ART (CD4 count >350 cells per μL)	$5·40	Point estimate	..	Eaton et al (2014)^34^	$14·04	Point estimate	..	Eaton et al (2014)^34^
Cost of health care for HIV-positive person not on ART (CD4 count 200–350 cells per μL)	$18·36	Point estimate	..	Eaton et al (2014)^34^	$49·68	Point estimate	..	Eaton et al (2014)^34^
Cost of health care for HIV-positive person not on ART (CD4 count <200 cells per μL)	$68·04	Point estimate	..	Eaton et al (2014)^34^	$180·36	Point estimate	..	Eaton et al (2014)^34^
Cost of end-of-life care	$54·00	Point estimate	..	Eaton et al (2014)^34^	$172·80	Point estimate	..	Eaton et al (2014)^34^
Cost of ART initiation	$52·92	Point estimate	..	Eaton et al (2014)^34^	$102·60	Point estimate	..	Eaton et al (2014)^34^

Costs are expressed in 2017 US$. PSA=probabilistic sensitivity analysis. CHiP=community HIV care provider. ART=antiretroviral therapy.

* Cost parameters with PSA distributions were varied in probabilistic sensitivity analysis, whereas cost parameters that constitute point estimates were not.

† Range specifies the parameters of the distribution used in the PSA.

‡ Since these unit costs were calculated by multiplying the per minute cost of a CHiP by the time spent per person covered (appendix 1 pp 6–7), the range varies the time component by + or −20%.

### Cost-effectiveness and budget impact

A probabilistic approach to cost-effectiveness analysis was applied by attaching costs and disability weights to each retained simulation either as point estimates or by varying key cost parameters (table 2, appendix 1 pp 8–9, 26), generating 1000 estimates of the intervention and counterfactual standard care costs and health outcomes (DALYs and new infections), in each of the 14 communities. All cost components listed in table 2 were included in the cost-effectiveness analysis. Disability weights were drawn from the Global Burden of Disease Study 2010 (appendix 1 p 26).^35^ Using a random ordering of the 1000 estimates in each community, we totalled costs and health outcomes across the 14 intervention communities, generating estimates of total cost, total DALYs, and total infections under the intervention and counterfactual approaches. DALYs and infections averted are presented in box plots with median and IQR values, and as percentage of infections and DALYs averted with the intervention compared with the counterfactual standard care. For each estimate of total cost and health outcomes (DALYs or infections), ICERs of the intervention compared with standard care were calculated as the difference in total cost between the intervention and counterfactual, divided by the difference in total health outcomes. Results are summarised as median ICERs and 95% credible intervals (CrIs). In calculating ICERs, PopART 2014–30 and PopART 2014–17 were independently compared with standard care. The three strategies were not incrementally compared with each other as PopART 2014–30 is our primary intervention scenario from a policy perspective to reach the UNAIDS 2030 Fast-Track target. Uncertainty in ICERs across parameter draws was summarised via a cost-effectiveness plane with a corresponding 95% credible ellipse.

We present two outcome measures from a health-care system perspective: cost per infection averted and cost per DALY averted. Future total costs and total health outcomes were discounted at an annual rate of 3%.^36^, ^37^

Cost-effectiveness acceptability curves are presented to summarise the probability of the intervention being cost-effective at different thresholds of cost per DALY averted. Model projections were used in a budget impact analysis, projecting the undiscounted costs of the intervention compared with counterfactual standard care up to 2030 in the PopART 2014–30 scenario. Average cost per person per year of delivering universal HBCT, linkage to care, promotion of ART adherence, and VMMC via CHiPs over the trial period was estimated by dividing the total projected CHiP costs for these components by the simulated population older than 14 years.

As a sensitivity analysis, we evaluated the effect of discount rate and time horizon on ICERs by varying the discount rate at 1% and 8%, and the time horizon in increments of 5 years (2035 and 2040). We also present ICERs by trial arm as a further sensitivity analysis to identify potential differences due to ART eligibility for 2014–15. Additionally, we did a one-way parameter sensitivity analysis, in which we individually varied four epidemiological parameters not varied in the PopART-IBM calibration (misreporting of partnership formation rates, relative infectivity of acute and early HIV infection compared with CD4 ≥500 cells per μL, CD4 progression when on ART but virally unsuppressed, and partnership formation between trial communities and immediate neighbourhoods) and three cost parameters relating to the PopART intervention (cost per person per year on ART, cost per VMMC, and per-min cost of CHiPs; appendix 1 pp 28–29) while holding others at their central estimate in the PopART-IBM or point estimates of costs (appendix 1 p 9). Resulting effect on ICERs is summarised in tornado plots.

### Role of the funding source

The funder of the study had no role in study design, data collection, data analysis, data interpretation, or writing of the report.

## Results

**Figure 1:**
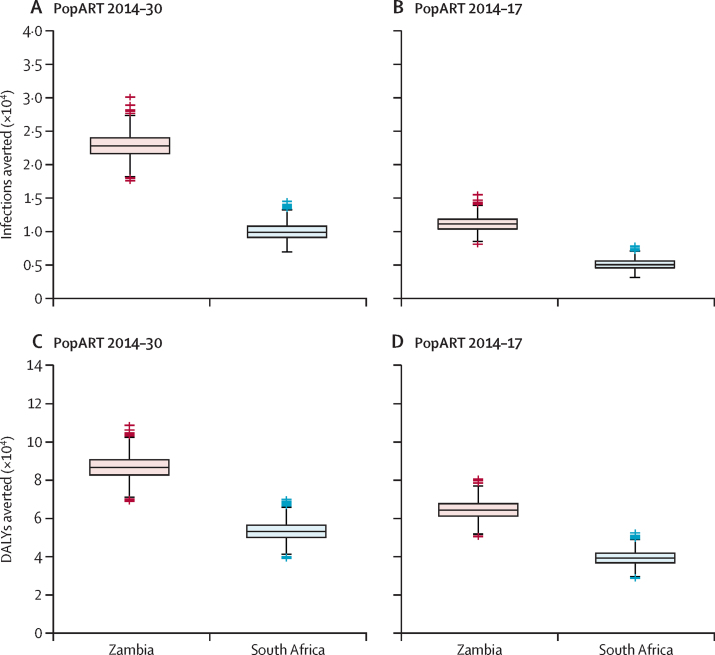
Health effects of the PopART intervention under different scenarios Box plots show the median (IQR) for 1000 retained simulations of new HIV infections and DALYs averted in Zambia and South Africa in 2014–30. Outliers were calculated as datapoints greater or less than 1·5× the IQR from upper and lower IQR values. (A and C) PopART 2014–30 scenario: PopART intervention implemented in annual rounds between 2014 and 2030. (B and D) PopART 2014–17 scenario: PopART intervention implemented in three annual rounds between 2014 and 2017 and then discontinued up to 2030. DALY=disability-adjusted life-year. The simulated mean annual population covered in 2014–30 (accounting for population growth) was 341 323 in Zambia and 165 852 in South Africa.

The greatest HIV incidence reductions were projected (appendix 1 p 10) in the PopART 2014–30 scenario, in which the intervention is implemented annually for 17 years. By contrast, in the PopART 2014–17 scenario, an initial decrease in incidence during three annual rounds of PopART between 2014 and 2017 is followed by an increase in incidence from 2019, almost approaching incidence in the counterfactual standard care scenario by 2030. In the PopART 2014–30 scenario, a median of 22 769 (IQR 21 599–23 975; 48·7%) new HIV infections in Zambia and 9805 (9069–10 729; 38·6%) new HIV infections in South Africa could be averted when compared with standard care. Additionally, a median of 86 413 (82 547–90 452; 39·8%) DALYs in Zambia and 52 961 (39·5%) DALYs in South Africa could be averted (figure 1, table 3). In the PopART 2014–17 scenario, 11 110 (10 349–11 832; 23·7%) new HIV infections in Zambia and 5026 (19·8%) new infections in South Africa could be averted; and 64 305 (61 155–67 601; 29·6%) DALYs in Zambia and 39 239 (36 773–41 744; 29·2%) DALYs in South Africa could be averted, compared with standard care. Projected population sizes were notably different between the countries, with a mean annual population simulated in 2014–30 of 341 323 in Zambia and 165 852 in South Africa.

**Table 3:** Key cost, cost-effectiveness, health gain, and budget impact results by scenario

	**Zambia**		**South Africa**	
	PopART 2014–30	PopART 2014–17	PopART 2014–30	PopART 2014–17
**Cost**				
Mean cost per person per year during the trial period*	$6·53 (0·29)	$6·53 (0·29)	$7·93 (0·16)	$7·93 (0·16)
**Cost-effectiveness**				
ICER: cost per HIV infection averted	$2111 (1827–2462)	$1318 (1098–1591)	$3248 (2472–3963)	$2236 (1601–2916)
ICER: cost per DALY averted	$593 (526–674)	$258 (225–298)	$645 (538–757)	$326 (266–391)
**Health gains and budget impact**				
Annual cost of PopART during the trial period (undiscounted)	$3·98 million (2014), $4·44 million (2015), $4·58 million (2016), $4·88 million (2017)	$3·98 million (2014), $4·44 million (2015), $4·58 million (2016), $4·88 million (2017)	$2·61 million (2014), $3·10 million (2015), $3·17 million (2016), $3·25 million (2017)	$2·61 million (2014), $3·10 million (2015), $3·17 million (2016), $3·25 million (2017)
Incremental cost (undiscounted)	$46·12 million (115·0%)	$12·67 million (31·8%)	$30·24 million (118·2%)	$9·89 million (38·7%)
Incremental HIV infections averted (undiscounted)	22 769 (48·7%)	11 110 (23·7%)	9805 (38·6%)	5026 (19·8%)
Incremental DALYs averted (undiscounted)	86 413 (39·8%)	64 305 (29·6%)	52 961 (39·5%)	39 239 (29·2%)

Costs are expressed in 2017 US$. ICERs are the median and 95% credible intervals for 1000 simulations. Other data are presented as the mean (SD) or absolute value (percentage of counterfactual standard care). Incremental values represent the difference between the intervention compared with counterfactual simulations. ICER=incremental cost-effectiveness ratio. DALY=disability-adjusted life-year.

* Includes home-based HIV counselling and testing, linkage to care, promotion of antiretroviral therapy adherence, and voluntary medical male circumcision, delivered by community HIV care providers to the population older than 14 years.

**Figure 2:**
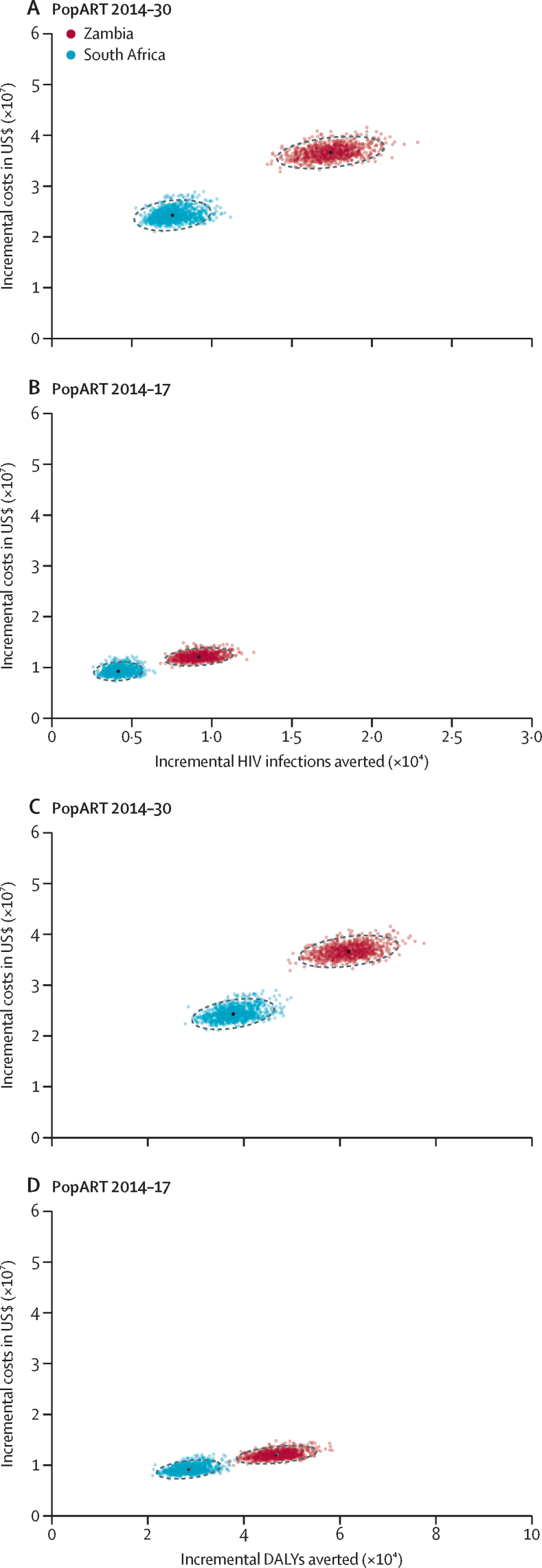
Cost-effectiveness planes Incremental cost-effectiveness ratios for PopART 2014–30 and PopART 2014–17 scenarios compared with standard care. Graphs show simulations, with median cost plotted against median effect. (A and C) Incremental costs and HIV infections or DALYs averted in the PopART 2014–30 scenario. (B and D) Incremental costs and HIV infections or DALYs averted in the PopART 2014–17 scenario. DALY=disability-adjusted life-year.

During 2014–17, the mean cost per person per year of delivering universal HBCT, linkage to care, promotion of ART adherence, and VMMC via CHiPs in the simulated population older than 14 years was $6·53 (SD 0·29) in Zambia and $7·93 (SD 0·16) in South Africa. The PopART 2014–30 scenario produced median ICER values of $2111 (95% CrI 1827–2462) per HIV infection averted in Zambia and $3248 (2472–3963) per HIV infection averted in South Africa; and $593 (526–674) per DALY averted in Zambia and $645 (538–757) per DALY averted in South Africa, compared with standard care (table 3). The entire ICER distribution for both HIV infections and DALYs averted implied PopART could avert more infections and DALYs than standard care, but at higher costs (figure 2). The ICER per HIV infection or DALY averted was lower in the PopART 2014–17 scenario with discontinuation of the intervention. In this scenario, PopART averted one infection at a cost of $1318 (1098–1591) in Zambia and $2236 (1601–2916) in South Africa, and one DALY at a cost of $258 (225–298) in Zambia and $326 (266–391) in South Africa, when outcomes were projected until 2030 (figure 2 and table 3). However, as shown by our projections (appendix 1 p 10), discontinuation of the intervention would result in increased HIV incidence after an initial decrease.

**Figure 3:**
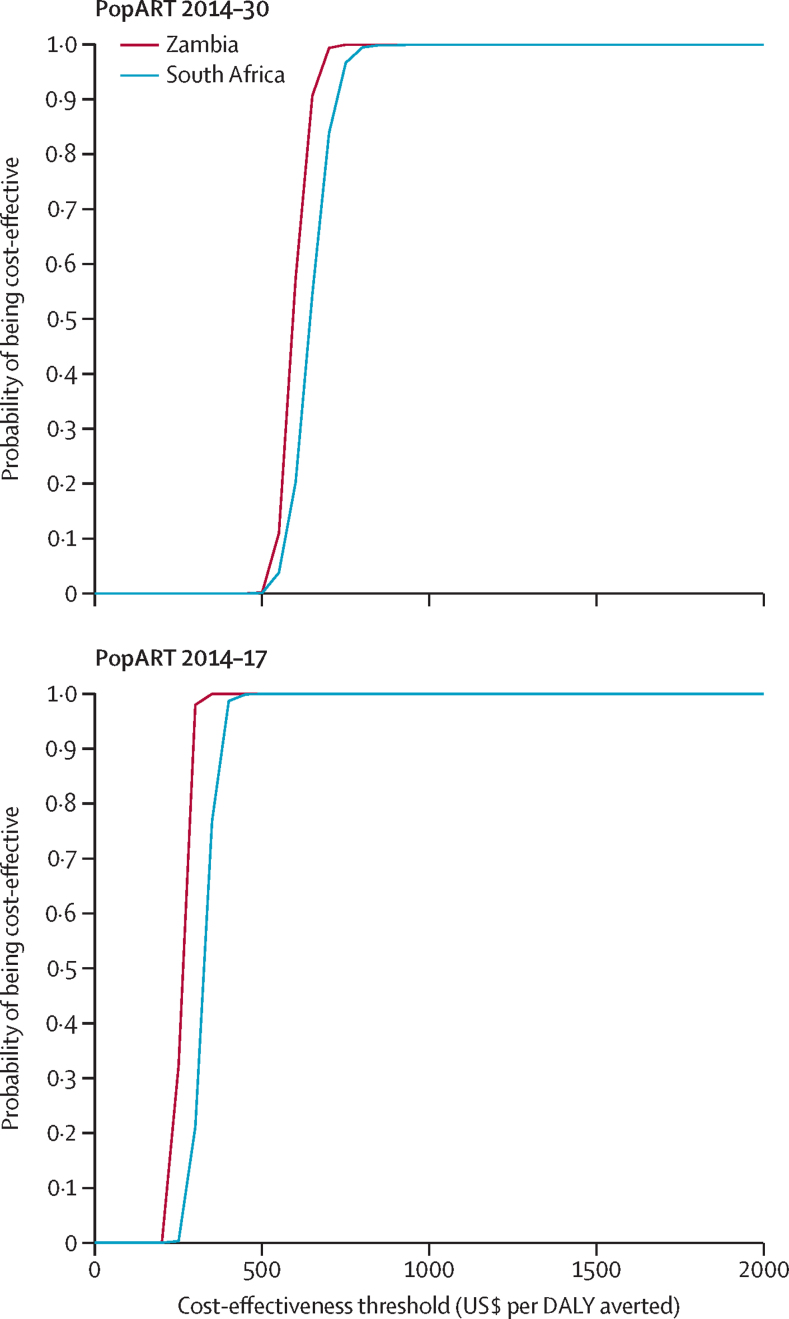
Cost-effectiveness acceptability curves by cost per DALY thresholds Cost-effectiveness acceptability curves represent the probability that the intervention is cost-effective across the simulations at specific thresholds of cost per DALY averted. DALY=disability-adjusted life-year.

Cost-effectiveness acceptability curves (figure 3) showed almost 100% probability of the intervention being cost-effective at thresholds greater than $700 per DALY averted in Zambia, and greater than $800 per DALY averted in South Africa, in the PopART 2014–30 scenario. In the PopART 2014–17 scenario, these thresholds were $350 per DALY averted in Zambia and $450 per DALY averted in South Africa.

**Figure 4:**
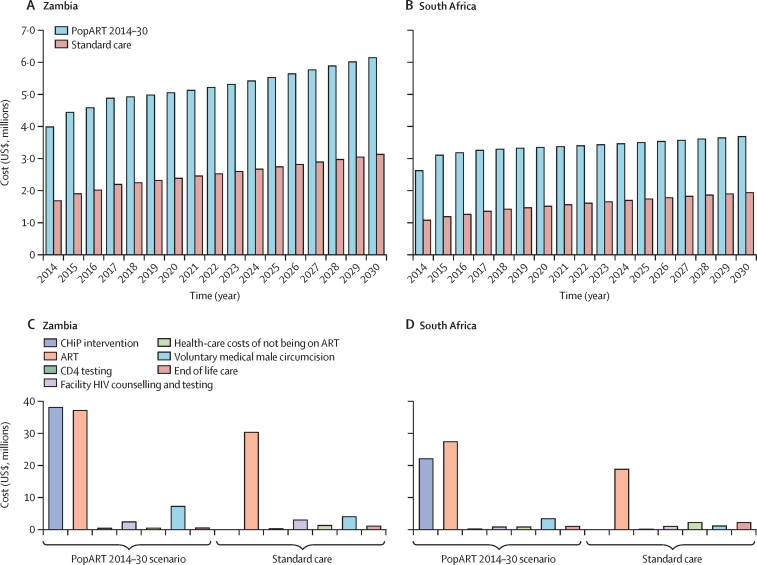
Budget impact of the PopART 2014–30 scenario Projected undiscounted annual cost (all cost components) in intervention communities and standard care (counterfactual) communities in the PopART 2014–30 scenario in Zambia (A) and South Africa (B). Projected undiscounted costs totalled for the period 2014–30 by cost component in Zambia (C) and South Africa (D). CHiP=community HIV care provider. ART=antiretroviral therapy.

During the trial period (2014–17), the annual costs of delivering HIV care, including the PopART intervention, for the simulated population older than 14 years ranged from $3·98 million to $4·88 million in Zambia and from $2·61 million to $3·25 million in South Africa (table 3). We projected these costs to increase over the simulation period in the PopART 2014–30 scenario (figure 4A, 4B). A similar trend was evident in the counterfactual simulating standard care. The larger cost increase in Zambia is most likely explained by population growth and an increasing number of people living with HIV on ART. The magnitude of differences in annual costs between countries can be attributed to different population sizes. The estimated undiscounted incremental cost of implementing the PopART intervention for the entirety of 2014–30 over standard care was $46·12 million (115·0%) in Zambia and $30·24 million (118·2%) in South Africa (table 3). We identified CHiPs and ART to be the two most costly components. These components would incur similar costs in Zambia, whereas the cost of ART would exceed that of CHiPs in South Africa during 2014–30 (figure 4C, 4D).

Varying the discount rate in our sensitivity analysis resulted in small changes in the estimated ICERs (appendix 1 p 27). Increasing the model time horizon to 2035 and 2040 resulted in successively lower ICERs compared with the 2030 projections, as future health benefits and cost savings from reduced incidence have a greater effect than the cost outlay (appendix 1 p 28). Analysis by trial arm resulted in small changes in the estimated ICERs with slightly larger ICERs in arm B, which had ART according to CD4 thresholds in the first two simulation years (appendix 1 p 28). One-way parameter uncertainty analysis showed ART costs and the extent of misreporting of partnership formation to have the greatest effect on ICERs (appendix 1 p 25).

## Discussion

Universal HBCT, linkage to care, promotion of ART adherence, and VMMC can be delivered via CHiPs at scale at low cost, at $6·53 per person per year in Zambia and $7·93 per person per year in South Africa. PopART delivered over three rounds is cost-effective if policy makers have a cost-effectiveness threshold of at least $350 per DALY averted in Zambia and $450 per DALY averted in South Africa. Delivered in annual rounds for 17 years (2014–30), PopART is cost-effective at thresholds greater than $800 per DALY averted. Although programme costs are high for annual implementation up to 2030, they correspond to a maintained effect on HIV incidence, whereas lower costs for a 3-year implementation are offset by a non-sustained reduction in HIV incidence.

In comparison with other studies,^10^, ^11^, ^12^, ^13^, ^14^ our ICER estimates are lower, suggesting that the cost-effectiveness of combination prevention including HBCT and promotion of VMMC, in addition to ART, at the population level is greater than previously thought. Two studies on HBCT estimated ICERs of $860–1710 per QALY^13^ at increasing ART thresholds (up to a CD4 count <500 cells per μL), and an ICER of $1360 per DALY averted with universal ART.^11^ A third study concluded that a combination prevention intervention is cost-effective at willingness-to-pay thresholds greater than $1690 per DALY averted.^10^ ICER estimates for HBCT in terms of infections averted were markedly higher at $11 480^9^ and $8750–22 000.^11^ However, previous studies differ in several key features from our study, most notably the frequency of testing rounds every 6 months^12^ or every 2 years,^12^, ^14^ 4 years,^11^ or 5 years^13^ (*vs* annual rounds in the PopART trial) and projection over 10 years,^11^, ^12^, ^13^ 14 years,^10^ or 20 years.^14^ Generally, cost-effectiveness analyses are sensitive to future incidence projections;^14^ therefore differences in the epidemiological models might explain part of the variations in estimates. Studies also differed in the effectiveness of the intervention, as measured by its coverage, linkage to care, coverage of ART, and viral suppression. Three studies used data from rural communities,^11^, ^12^, ^13^ and the low population density and small scale of delivery might have resulted in higher implementation costs compared with the urban and peri-urban communities in PopART. Additionally, the reduction in drug costs in the past 10–15 years could be important in explaining the lower cost-effectiveness in previous studies. ICER estimates have been shown to vary with the costs of ART drugs and care, particularly if there is no eligibility criteria for ART initiation.^11^, ^13^ Previous studies assumed the per person per year costs of ART at $682^13^ and $565,^11^ which are higher than our estimated costs of ART at $212 (Zambia) and $315 (South Africa).

Is a combination intervention including UTT worth implementing as part of standard of care in high prevalence settings? Our findings suggest PopART should be considered for implementation or integration into existing government programmes. Compared with facility-based care provision, PopART could generate substantial health gains. These health gains would be due to reduced incidence, resulting from improved linkage to care and retention in care of previously undiagnosed and untreated HIV-infected people, and the success of ART in extending lifespan nearly up to that of uninfected people.^38^, ^39^ However, the annual cost of PopART is not outweighed by the cost savings to the health-care system that arise from averting new infections. The decision to invest in PopART depends ultimately on the cost-effectiveness threshold of policy makers and donors supporting HIV programmes, with such a threshold being the decision criteria for when benefits from a new intervention are considered sufficient in comparison with its costs.^40^ Policy makers and donors in high prevalence settings can use our cost-effectiveness acceptability curves to identify the likelihood of PopART being cost-effective for their threshold. Estimates published in 2016 of cost-effectiveness thresholds had a wide range, reflecting the data constraints and methodological difficulties in reliably estimating thresholds.^40^ Donors' thresholds might also differ from those of a country's policy maker due to different budget constraints and valuations of health benefits based on funding priorities. In cases in which PopART might not lie within a country's threshold, donors have a role in subsidising its implementation to the point that it does become cost-effective. Such an approach does not crowd out domestic financing, while ensuring a fair and sustainable allocation of aid with time as countries move to lower-middle-income and middle-income status and donors become more selective in the programmes they finance.^41^

The affordability of PopART is an important consideration. Although the estimated average costs of the intervention per person covered are low, they sum up to a considerable amount when projected for the total population older than 14 years. Population growth needs to be factored in, because expenditures on combination prevention with UTT will increase simply due to the increasing population that needs to be covered by the intervention. The optimal frequency of testing rounds subject to the available budget might be an important consideration. We show that three rounds cannot sustain the initial reduction in incidence, and numbers of new infections approach those under standard care after 13 years. A modelling study of an HBCT campaign in western Kenya showed that in 20 years, DALYs averted could be maximised with a so-called front-loaded scenario, whereby four testing rounds are delivered in years 1, 2, 4, and 8, compared with a scenario of five equally spaced rounds every 4 years.^42^ More research is needed exploring alternative and sustainable approaches to HIV test provision, including initial door-to-door testing followed by mobile or hub-based testing, self-testing,^43^, ^44^ or potentially decreasing the frequency of door-to-door testing.

This study has limitations. Technological innovation, prices of testing and treatment, guidelines, efficacy of standard care, behaviour of individuals, population dynamics, valuation of health outcomes, and many other factors are likely to change in 17 years and affect costs and benefits. Although we used detailed costing approaches to precisely allocate shared inputs when costing HIV care at health-care facilities and to separate the research and implementation costs of PopART, the likelihood of inaccuracies remains in some of our costs. Costs across communities also vary substantially, and the extent to which our results are generalisable to other settings depends on their similarity to PopART trial communities, including HIV prevalence, and costs of hospitalisation and end-of-life care. The generalisability of results also depends on the scale and efficiency of implementation by public providers if adopted as part of a national programme. To address this limitation, we allowed for wide variations in crucial parameters in sensitivity analyses. In addition, if policy makers are considering scale-up to national levels, our estimates do not account for potential diminishing marginal returns from provision of HBCT to hard-to-reach populations. Migration between communities within and outside of the study is also likely to affect both benefits and costs, and it is difficult to ascertain whether and in what direction this might have biased our results.

Several objections are commonly put forward against large-scale, population-level combination prevention interventions. The yield or positivity rate of screening interventions targeted at the general population is low. Our findings on cost-effectiveness show that focusing on testing yield as an outcome measure is too simplistic. This measure does not recognise that combination prevention, as we have modelled, affects population health via several pathways, which are all captured in the overall DALYs and infections averted as measures of the ultimate health gain of the intervention. Another objection is that an unknown proportion of individuals who have newly tested as HIV-positive are retested. However, this is not necessarily a waste of resources because studies have shown that linkage to care is increased in individuals who had previously dropped out of care.^14^, ^45^ Prevention interventions that prioritise specific subpopulations on the basis of risk factors are often more cost-effective than interventions that target the general population, at least in the short term.^46^ However, targeted interventions often have only modest effect on population-level HIV incidence and mortality.^14^ Our results show that population-level strategies can be delivered at scale at low per-person cost and generate substantial health gains. The cost-effectiveness depends on the specific thresholds for health gains of policy makers and donors. The findings of our study should help to identify worthwhile investments into HIV interventions and support epidemic control.

## Data sharing

The HPTN 071 (PopART) data sharing policy is provided in appendix 2.
