# Supplementary material for: Cost and cost-effectiveness of a universal HIV testing and treatment intervention in Zambia and South Africa: evidence and projections from the HPTN 071 (PopART) trial
Source: Lancet Glob Health. 2021 Mar 12;9(5):e668–80. doi: 10.1016/S2214-109X(21)00034-6 (PMC8050197; doi:10.1016/S2214-109X(21)00034-6)
Supplement: Supplementary appendix 1 [file mmc1.pdf]

THE LANCET
Global Health

Supplementary appendix 1

This appendix formed part of the original submission and has been peer reviewed. We post it as supplied by the authors.

Supplement to: Thomas R, Probert WJM, Sauter R, et al. Cost and cost-effectiveness of a universal HIV testing and treatment intervention in Zambia and South Africa: evidence and projections from the HPTN 071 (PopART) trial. *Lancet Glob Health* 2021; published online March 12. https://doi.org/10.1016/S2214-109X(21)00034-6.

Supplementary Appendix

Cost and cost-effectiveness of a universal HIV testing and treatment intervention in Zambia and South Africa: Evidence and projections from the HPTN 071(PopART) Trial

Ranjeeta Thomas, William Probert, Rafael Sauter, Lawrence Mwenge, Surya Singh, Sarah Kanema, Nosivuyile Vanqa, Abigail Harper, Ronelle Burger, Anne Cori, Michael Pickles, Nomtha Bell-Mandla, Blia Yang, Justin Bwalya, Mwelwa Phiri, Kwame Shanaube, Sian Floyd, Deborah Donnell, Peter Bock, Helen Ayles, Sarah Fidler, Richard Hayes, Christophe Fraser, Katharina Hauck

On behalf of the HPTN 071 (PopART) study team

Modelling ... 2
Overview of the PopART individual-based simulation model (PopART-IBM) ... 2
Inference framework ... 4
Cost and Cost-effectiveness Analysis ... 5
Cost of delivering the CHiPs intervention ... 5
Unit cost of CHiPs – model inputs ... 5
Average cost per-person per-year of the CHiPs intervention ... 7
Cost per-person per year on ART at healthcare facilities ... 7
Cost-effectiveness analysis (CEA) ... 8
One-way Parameter Sensitivity Analysis ... 9
Figure S1: Projected mean HIV incidence in the PopART-IBM 2010-2030 under two scenarios of the PopART intervention, and standard care without the CHiPs intervention. ... 10
Figure S2 A-N: HIV care cascades by PopART trial community ... 24
Figure S3: One-way Parameter Sensitivity Analysis ... 25
Table S1:Disability weights ... 26
Table S2: CHiPs Time Spent Per Person ... 26
Table S3: Cost-per-person-per year on ART (US$) ... 26
Table S4: ICERs at discount rates of 1% and 8% ... 27
Table S5: ICERs by model time horizons ... 28
Table S6: Results by trial arm ... 28
Table S7: Parameter ranges for one-way sensitivity analysis ... 28
References ... 30

Modelling

Overview of the PopART individual-based simulation model (PopART-IBM)

The PopART-IBM has a modular structure, with components that each affect the predicted dynamics in trial communities. These include geographic structure, demographics, heterosexual partnerships, HIV natural history, HIV transmission, and both the HPTN 071 (PopART) intervention and a background HIV-care cascade. Both the PopART intervention and the background HIV-care cascade include HIV testing, HIV care and ART, and and voluntary male medical circumcision (VMMC). The model introduces HIV into the simulated population between 1970-80 and projects outcomes until the specified end year. The model seeds HIV in a small random sample of 18-30 year olds for each year for 5 years following the date of HIV introduction. The size of the seeding population is varied in the calibration of the model. Each calendar year in the model is divided into 48 discrete timesteps. At each timestep a series of processes are implemented, for example – deaths from natural causes, followed by entry of new individuals into the modelled adult (aged >14) population and then new births, new becoming HIV-positive,HIV infections, starting ART initiations, and and having a new sexual partnerships. Intervention related activties such as visits by Community HIV Care Provider (CHiPs) teams, improved ART initiation and increased uptake of VMMC as a consequence of CHiPs visits only occur at and after specified time periods which correspond to the intervention scenarios modelled. All individuals in the model have a set of associated characteristics defined at their entry into the model. Over their life course individuals experience different events including formation and dissolution of partnerships, ageing, HIV testing and death. Within the model individuals form partnerships assortatively by age and inherent level of sexual behaviour risk. Mixing by age is determined by age-mixing matrices derived from HPTN 071 (PopART) trial data, while mixing by risk is governed by a parameter, determined during calibration, that ranges from fully assortative to fully proportionate mixing. The events experienced by an individual are determined by their characteristics, characteristics of the wider population, and time. Code for the model is available under the GNU General Public License 3·0 at: https://github.com/BDI-pathogens/POPART-IBM.

Geographic structure and partnerships involving people outside the community: The PopART-IBM is constructed using "patches", where an "inside" patch represents the trial community, and an "outside" patch consists of the immediate neighbourhood of equilvalent size and characteristics to the inside patch. Between-patch sexual partnerships may form between individuals in different patches but HIV testing and treatment occurs according to the patch from which that individual belongs. The majority of input parameters are the same in both patches, however the inside patch includes the PopART intervention and CHiPs household visits in the intervention simulations, which improve ART iniation and uptake of VMMC compared to non-trial

areas. These are based on data that were collected from community members during household visits by CHiPs.The PopART-IBM does not currently model migration.

Population demographics: The PopART-IBM simulates a growing population of approximately comparable size to a trial community (modelled as the "inside patch"). The population size in each community changes over time based on differences in fertility and mortality rates taken from country-specific United Nations Population Division (UNPD) 2015 World Population Prospects estimates and the medium variant of UNPD projections of these measures into the future. UNPD mortality rates for each country are adjusted to remove HIV-related deaths since the PopART-IBM explicitly models the HIV epidemic and HIV disease progression. Adults (aged >14) form the primary population. The threshold of above 14 years is intended to capture the majority of time of exposure to HIV risk through sexual activity. Mother to child transmission is not modelled. All individuals reaching adulthood begin with a set of epidemiological characteristics which are updated as events happen to individuals (for example change in HIV status). These characteristics include sex, date of birth, risk group, HIV-status etc. At each timestep in the model, the number of new births who survive to adulthood is calculated. An equivalent number of adults are then modelled 14 years later. Date of birth is then specified as the timestep at birth. All individuals enter the adult population as HIV-negative. A certain proportion of the male population is assumed to undergo traditional male circumsision (TMC) at birth as determined by community-specific prevalence from all rounds of the Population Cohort. While a number of reviews (e.g.Weiss, Quigley [1]) have found that traditional male circumcision (TMC) is associated with lower HIV prevalence in many settings in sub-Saharan Africa, there is increasing evidence that in some settings TMC is less protective, or unprotective against HIV acquisition[2]. Due to this ambiguous evidence, cross-sectional data from the PopART baseline survey was used to assess the extent to which TMC, as practiced in the PopART communities, was associated with HIV prevalence. In a more extended analysis (to be published) we found no evidence for reduced HIV prevalence in men who underwent TMC, compared to uncircumcised men, in either South Africa or Zambia. VMMC is assumed to offer a 60% reduction in susceptibility,[3,4] while traditional male circumcision (TMC) is assumed to offer no protection[5]. The Population Cohort (PC) is a random sample of ~2500 individuals, aged 18-44, per trial community within which the primary endpoint of the trial was measured.

Health and care states: Individuals are modelled as having an HIV status, an ART status, a CD4 category, and a set-point viral load (SPVL). HIV status may be negative or positive. If HIV positive, an individual may be in either the phase of acute and early HIV infection (AEHI) or the chronic phase of HIV infection. Individuals may start ART if they have an HIV positive test result or if they become acutely unwell following the development of AIDS (defined as CD4 < 200 within the model). Individuals initiating ART have a 2-month period when they are not fully virally suppressed. After this early ART period individuals either stay virally suppressed (zero chance of transmitting HIV), have poor adherence and become virally unsuppressed (50% reduction in relative risk of transmitting

HIV compared to not being on treatment), or drop out of care. SPVL is drawn from a distribution when an individual becomes HIV positive and follows Fraser et al., (2007)[6]. CD4 category is subsequently drawn following a distribution based on the individual's SPVL. The rate of CD4 progression also depends on SPVL. Both the probability of being in a given CD4 category and the rate of CD4 progression follow data from the AIDS Therapy Evaluation in the Netherlands study (ATHENA) cohort, as published in Cori and Pickles et al., (2015).[7] An individual's infectivity is modified by their SPVL, CD4 category, whether they are in AEHI or not, and ART status, as well as by their sex.

Interventions: The PopART-IBM includes HIV testing, ART, and VMMC as part of both the CHiPs intervention and the background care cascade. The CHiPs intervention is assumed to follow coverage as observed in the trial, stratified by age and sex, with each visit assumed to include HIV testing. The probability of an individual having an HIV test in the background cascade is estimated with two parameters in the calibration, one giving the probability of an HIV test from 2000-2006 and a second specifying the annual probability from 2006 onwards. Following an HIV positive test result, if an individual is eligible to start ART (according to national guidelines or guidelines of the trial arm), then time until ART initiation follows an exponentially distributed time in the background cascade that is estimated in the calibration. Time until initiating ART following a CHiPs visit is distributed according to a biexponential distribution estimated using data from the trial, so that individuals are divided into fast and slow initiators as seen in the data. Data shows time to ART initiation reduced over the time of the study from 9-3 months. Following an HIV negative test result, men are offered a VMMC procedure, with an acceptance probability modelled as 40%. VMMC is only offered to people who are not already circumcised.

Counterfactual simulations: Counterfactual simulations are included as comparisons. In a counterfactual the same epidemic is simulated both with and without the intervention in question, in this case the intervention being the CHiPs intervention of HPTN 071 (PopART). Counterfactual simulations use the same parameters (including random seed) apart from PopART-related parameters. Because the same random seed is used in counterfactual simulations, the epidemic will be identical in the counterfactual simulation as in the non-counterfactual simulation up until the start of the simulated trial.

Inference framework

The PopART-IBM is calibrated using Approximate Bayesian Computation (ABC). ABC is a suite of algorithms that approximate a conditional probability density function, as described in Beaumont, Cornuet [8]. Calibration involves generating a large range of candidate parameter sets and simulating an epidemic using each parameter set to determine the level of concordance between observed data and the simulated analogues of these data. A distance measure compares simulated and observed

summary statistics. A simulated projection, for a given PopART-IBM input parameter combination, is accepted if the distance measure is below a predefined threshold. The resulting distribution of the model projections is proportional to the posterior distribution given the data and the model. For each community, 1000 parameter sets out of several hundred thousand that produce simulation results with the highest concordance with the data are retained. We use the ABC algorithm suggested by Lenormand, Jabot [9] called the adaptive PMC-ABC algorithm (see appendix in Lenormand, Jabot [9]) with a Eucledian distance measure. As summary statistics, we use the following data, all stratified by sex- and 5-year-age-group, to parameterise the PopART-IBM for each community:

- HIV prevalence from regional historical data (3 rounds of Demographic and Health Survey (DHS)[10] data in Zambia; 4 rounds of Human Sciences Research Council (HSRC) data in South Africa).
- Community-specific HIV prevalence from the trial (from the final CHiPs round and from 4 PC rounds)
- The community-specific proportion of HIV+ individuals aware of their HIV status (from 3 CHiPs rounds in Zambia, CHiPs round 3 in South Africa, and from 4 PC rounds in South Africa).
- The community-specific proportion of individuals on ART among those aware of their status (from 3 CHiPs rounds in Zambia, CHiPs round 3 in South Africa, and from 4 PC rounds in South Africa).
- The community-specific proportion of individuals virally suppressed among those on ART (at 24 months after trial start from the Population Cohort).

Age groups used in the calibration are in 5-year intervals from 15 to 60, that is: 15-19, 20-24, 25-29, … , 55-59, and >60. The PopART-IBM is calibrated to each community. The HIV care cascade under each of the modelled scenarios for the following key indicators are presented in Figures S2A-N. They highlight the gains made from PopART in two key indicators - the projected proportion of people living with HIV (PLHIV) aware of their HIV status (the entry point into the modelled HIV care cascade) and proportion of individuals on ART among those aware of their HIV status.

Cost and Cost-effectiveness Analysis

Cost of delivering the CHiPs intervention

Unit cost of CHiPs – model inputs

Costs of HBCT, linkage-to-care, promotion of ART adherence and VMMC through CHiPs are calculated using data generated by implementing the intervention during the trial. The trial covered the entire population in the communities regardless of age. Therefore these unit costs, calculated

using trial data are representive of delivering the intervention to all ages and are used as inputs in the PopART-IBM which simulates only the populaiton aged >14.

Costs were estimated under mutually exclusive categories of personnel, supplies, equipment, transport, facilities and and overheads. To determine the time taken for specific activities, a detailed time-and-motion (TAM) study was conducted in both countries in 2018. Randomly selected teams of CHiPs were shadowed over 2 days to observe the time in minutes spent on specific activities and tasks. In total 32-person days of data were collected across the 14 trial intervention communities. These data are used to calculate the average time (in minutes) it takes to deliver HBCT to one individual seen by a team of CHiPs (2 workers), broken down by specific activities, e.g. time taken for counselling, the first and confirmatory HIV tests, but also administrative tasks such as training, travelling and record keeping. The TAM allowed exclusion of the costs for all those activities for which benefits are not projected by the PopART-IBM, and not considered by the CEA, such as those related to screening for tuberculosis (TB)and sexually transmitted diseases (STDs). The costs of the CHiPs intervention also exclude costs of facility based HIV testing, HIV care delivered at the heatlhcare facilities, and the costs of ART and other medications. In addition, research time costs for activities such as informed consent could also be excluded. Thus, our costing approach is an operational costing model, omitting research-related costs. From the TAM we calculated average time spent on administration and travel per person covered in the population, offering the intervention, and for those taking up HIV testing and receiving either an HIV-positive or HIV-negative result. The CHiPs time spent per-person on the major activity categories is provided in table S2.

Personnel costs (salaries including benefits) for the CHiPs and the intervention management cadre were taken from trial human resource data and from financial status reports and converted to per minute costs of CHiPs personnel. CHiPs equipment costs were also extracted from study data and electronic data capture devices, rucksacks, bags, clothes etc. Costs of HIV test kits were based on study purchase prices and include the cost of Determine test kits and the additional cost of UniGold confirmatory test kits for those testing HIV-positive, costs of supply chain and supplies for conducting HIV tests.

The basic cost per-person per-year (pp/py) covered by CHiPs in the trial population was calculated by multiplying the per minute cost of a CHiPs team by the time spent per person covered on administration and travel, to which the CHiPs cost per-person covered in the population (regardless of type of client or age) for training, administrative overheads, stationary and consumables, facilities and equipment were added . These basic costs which represent a form of fixed cost of service delivery is incurred for the entire population covered by CHiPs, including persons who could not be contacted by the CHiPs, or persons refusing the intevention. In addition, cost of time spent on offering the intervention to individuals, cost of time spent on conducting an HIV test and the confirmatory test, linkage-to-care, promotion of ART adherence and VMMC, costs of HIV test kits and

consumables were included to estimate the cost per client testing HIV-positive or HIV-negative. The basic cost pp/py of delivering the CHiPs intervention in the trial intervention communities was $5·08 in Zambia and $6·36 in South Africa. The cost pp/py offered the intervention and taking up HIV testing through CHiPs was $14·07 (Zambia) and $16·90 (South Africa) per HIV-positive client detected and $9·08 (Zambia) and $10·76 (South Africa) per HIV-negative client detected (See Table 2 in the main text).

Average cost per-person per-year of the CHiPs intervention

The average cost pp/py of the CHiPs intervention over the trial period was calculated as a weighted average of the above described unit costs, where the weights are the proportion of individuals who accepted the intervention, who accepted an HIV-test and were tested HIV-positive and HIV-negative as modelled by PopART-IBM. The weights differ each year of the projection because of differences in key parameters of the model, including uptake of the intervention, incidence, yield, and population growth. The average cost pp/py of the CHiPs intervention was estimated by dividing the modelled total cost of delivering universal HBCT, linkage-to-care, promotion of ART adherence and VMMC through CHiPs to those aged >14 over the three years of the trial by the >14 population simulated in the PopART-IBM. The estimated average cost pp/py of delivering these CHiPs components of PopART between 2014-2017 for the simulated population of >14 was $6·53 (SD=0·29) in Zambia and $7·93 (SD=0·16) in South Africa.

Cost per-person per year on ART at healthcare facilities

Facility costing of ART services was conducted in all trial HIV health care facilities in Zambia and South Africa to estimate the cost per-person-per-year on ART. Facility surveys gathered information from financial status reports and interviews with service managers on costs relating to HIV treatment and care, on building and storage expenses, equipment, personnel and drug costs, as well as patient numbers in each facility. We estimated annual financial costs for resources used in the intervention. Each individual item was assigned an allocation cost ratio indicating how much of its services were related to HIV care. The allocation ratios were determined through a variety of methods depending on cost item, for example salary costs were allocated via interviews with service managers, building costs via a measurement of building space and their usage for HIV and non-HIV related care, and shared equipment such as laboratories via sample surveys of HIV and non-HIV related activities.

Building and storage costs were estimated through measurements collected in the facility-level surveys. Rooms for HIV care included waiting rooms, those for voluntary counselling and testing, adherence support, medical male circumcision, care for adolescents and adults, pre-ART care, care for HIV+ patients, electronic data capture, pharmacies for storage and dispensing, patient records, laboratory, and administration. In some cases, clinics were built several years ago and therefore, building costs only covered rental or remodelling costs. We assumed an economic life of 35 years

and a furnishing rate of 10%. The base year cost was therefore the sum of the annual cost and the furnishing cost multiplied by the allocation ratio.

Equipment costs included acquisition and maintenance costs for lab and medical supplies, vehicles, stationary/office supplies, utilities, security and communication costs (i.e. phones, internet, etc.). We assumed an economic life of 3 years. The annual base year cost was then the annual cost multiplied by the allocation ratio. Where missing, estimates were found through secondary sources such as WHO choice.[11]

Personnel costs included amount spent on staff salaries, benefits, and where applicable, training costs. Salaries and benefits came from the facility-level surveys and were determined by a standard salary scale provided by the facilities and the districts. There were separate salary benefits for non-medical staff (i.e. guards, cooks, cleaners) and medical staff (i.e. nurses, medical staff). The annual cost was the gross yearly salary plus the benefits and the base year cost is the annual cost multiplied by the allocation ratio.

Pharmaceutical costs included only ARV drug costs. The numbers of each type of ARV drug dispensed were collected from the facility-level survey by reviewing pharmacy records at each facility. Unit prices were collected from the Clinton Health Access Initiative reference price list[12] and the total annual cost was calculated by multiplying the total number of bottles dispensed and the unit price.

To calculate the unit cost per-person-per year on ART we summed the annual total costs of building, equipment, supplies, personnel, and ARV costs and divided by corresponding patient numbers. Thus, a unit cost per-person-per-year on ART was estimated for each health facility (Table S3). Using the variation in facility costs within each country, the mean and corresponding parameters of a gamma distribution were calculated and used in the cost-effectiveness analysis. The one extreme outlier in South Africa (community 21) is explained by very low patient numbers and was excluded as an outlier when generating the mean cost and parameters of the gamma distribution for the cost-effectiveness analysis.

Cost-effectiveness analysis (CEA)

The PopART-IBM is calibrated to each trial intervention community. For each of the 14 intervention communities, 1000 parameter sets that meet the criteria of the calibration method described earlier are retained for the CEA. In the CEA, costs are attached to each simulation either as point estimates or by varying key cost parameters (as per Table 2 in the main text). Costs do not vary in the analysis by community, but by simulation with random draws from specified distributions (see Table 2 in the main text). Total costs and effectiveness (DALYs and infections averted) are calculated by summing

random draws without replacement from the 1000 simulations in each community. Results are summarized as median ICERs and 95% credible intervals over these simulations.

One-way Parameter Sensitivity Analysis

We independently varied four epidemiological parameters not varied in the PopART-IBM calibration and three cost parameters to assess one-way parameter uncertainty (Table S7). In all cases the "best fitting" parameter set according to the PopART-IBM calibration method was selected and each of the four epidemiological and three cost parameters were varied individually. For each parameter, the mean over 10 model runs was used to estimate the ICER. Generated ICERs were compared with the ICER generated under the "best fitting" parameter set with costs at mean values from Table 2 (main text). Resulting impact on the ICER is presented as Tornado plots for each country.

In both countries cost per-person per year on ART has the greatest influence on the ICER (Figure S3), followed by uncertainty in the factor for misreporting of partnership formation rates. This is followed by the rate of relative infectivity of acute and early HIV infections in Zambia and relative rate of partnership formation between model patches in South Africa. The other parameters had very small influence on the ICER.

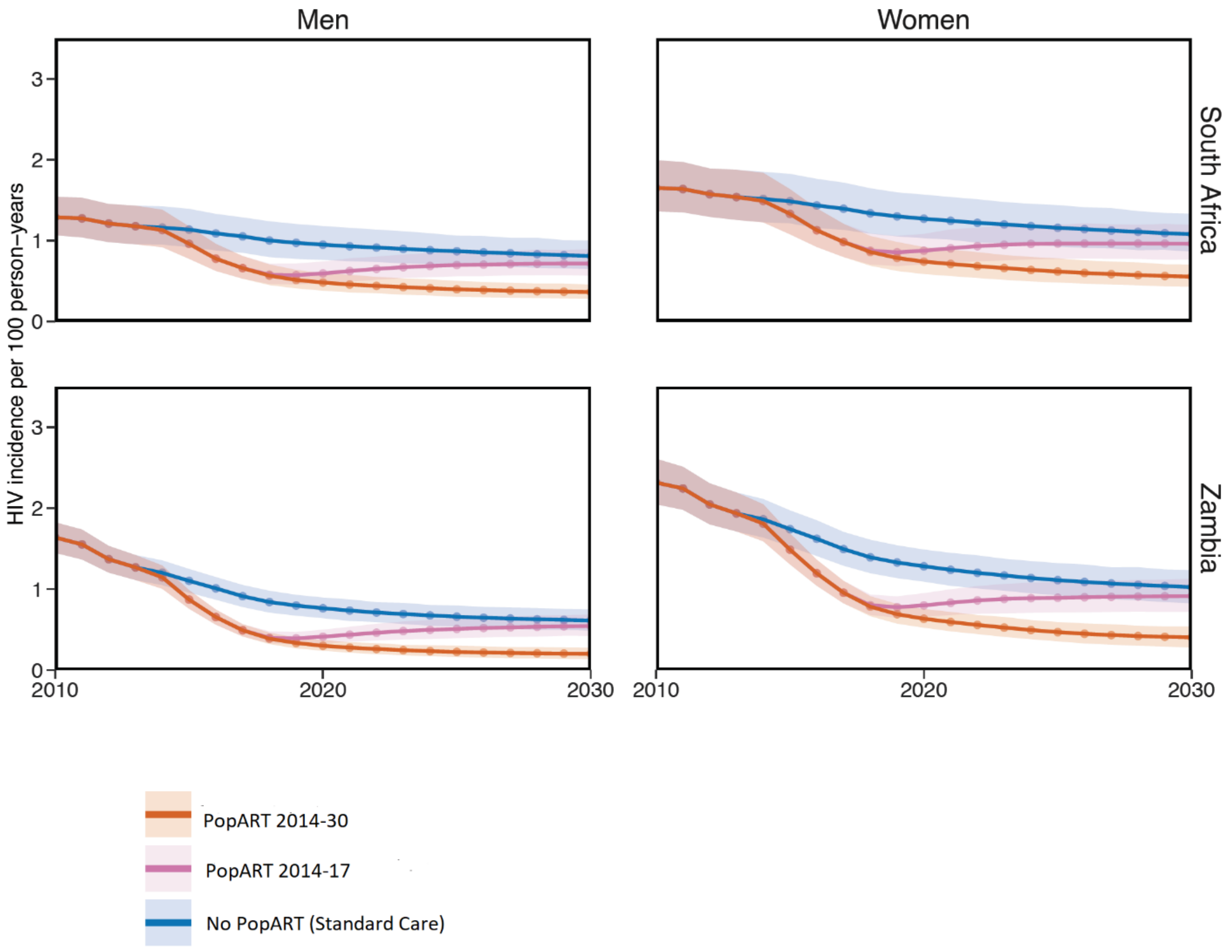

Figure S1: Projected mean HIV incidence in the PopART-IBM 2010-2030 under two scenarios of the PopART intervention, and standard care without the CHiPs intervention.

Notes for figure S1: HIV incidence by country and gender shown as mean, 2·5% and 97·5% quantiles of the mean incidence in the PopART-IBM calibration. PopART-IBM projects from the start of the HIV epidemic in the 1980s up to 2030 to show the trend and then divergence once the PopART intervention starts.

S2 A

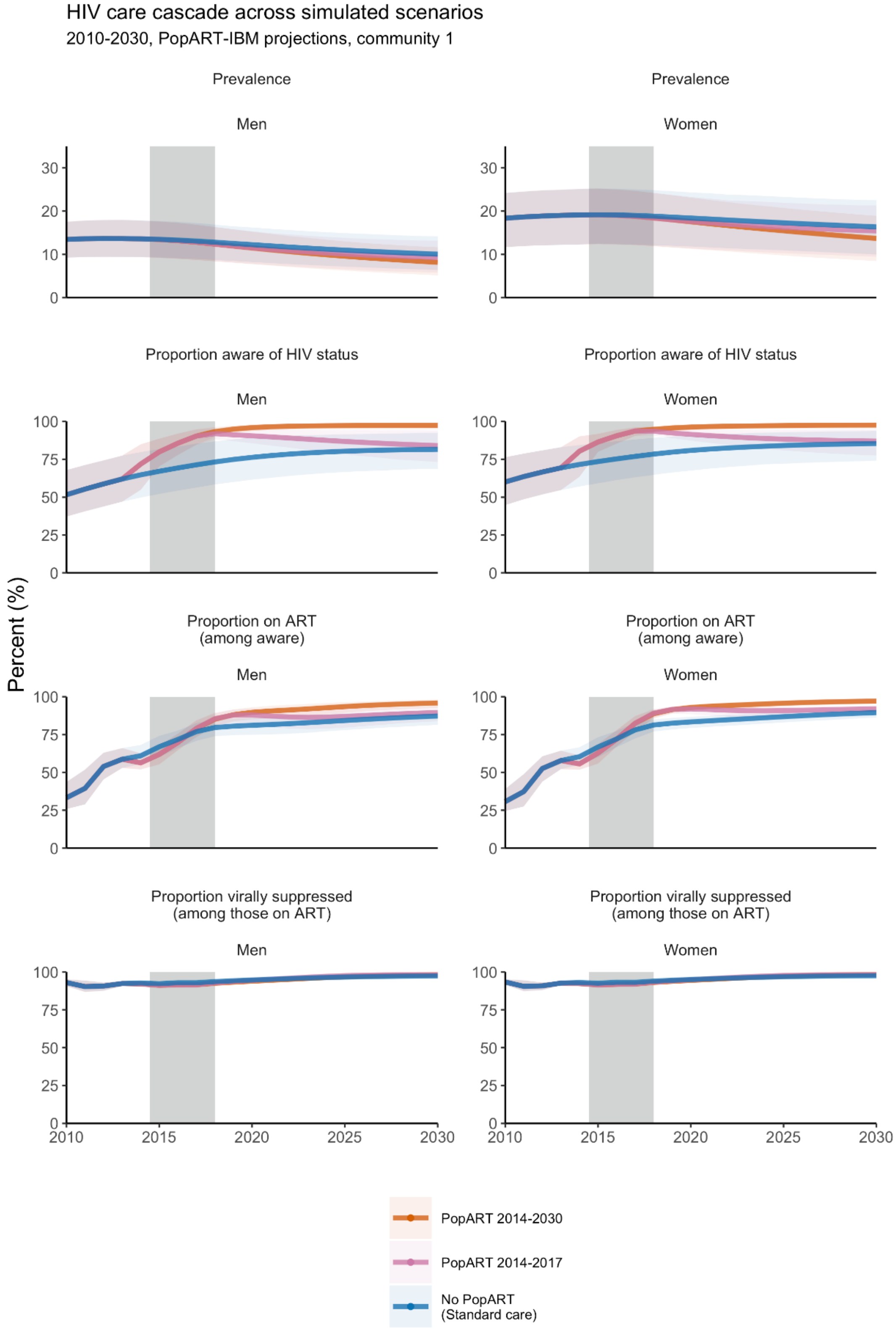

HIV care cascade across simulated scenarios
2010-2030, PopART-IBM projections, community 1
Prevalence
Men
Prevalence
Women
Proportion aware of HIV status
Proportion on ART
(among aware)
Proportion virally suppressed
(among those on ART)
Percent (%)
PopART 2014-2030
PopART 2014-2017
No PopART
(Standard care)

S2 B

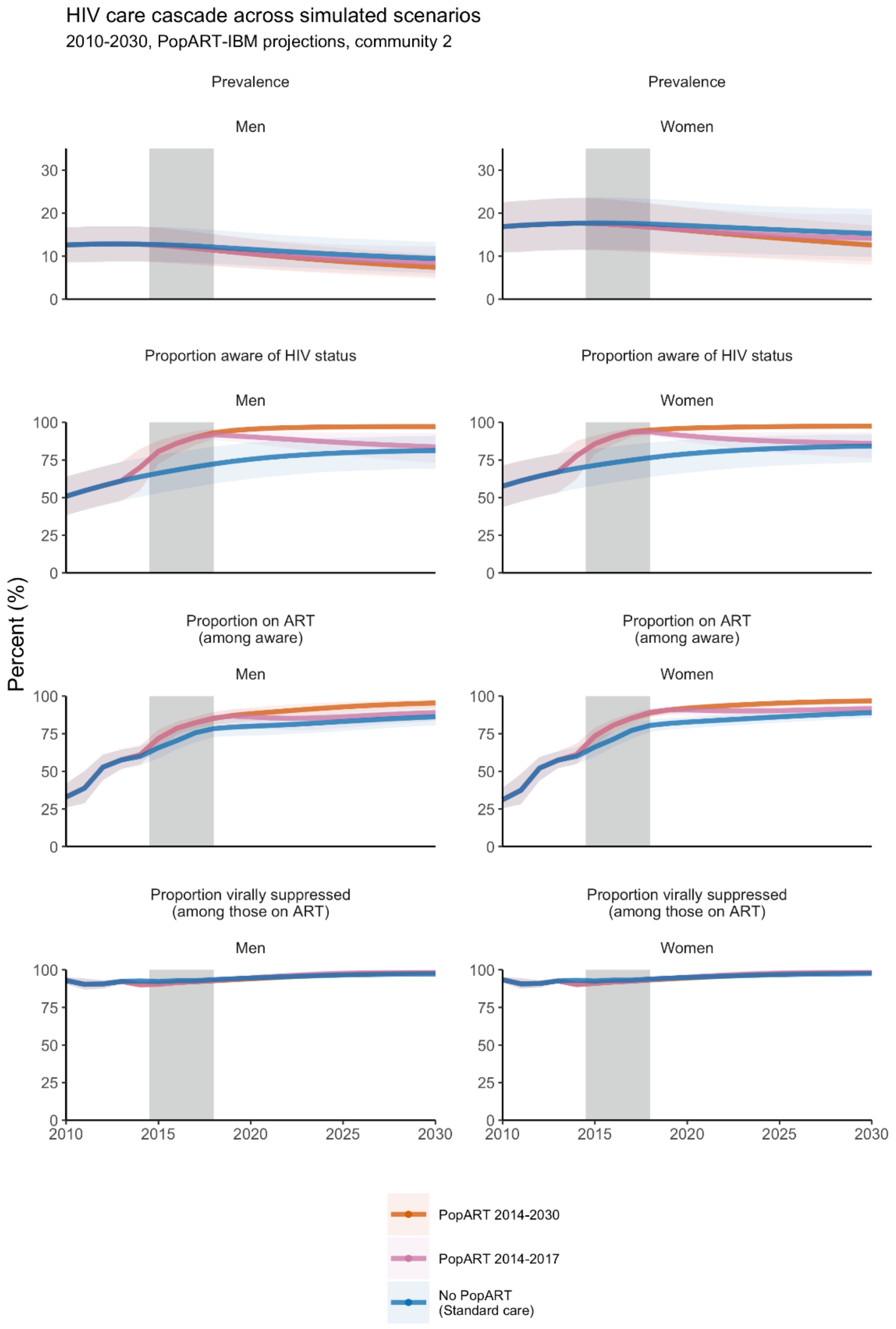
HIV care cascade across simulated scenarios
2010-2030, PopART-IBM projections, community 2
Prevalence
Men
Women
Proportion aware of HIV status
Proportion on ART
(among aware)
Proportion virally suppressed
(among those on ART)
Percent (%)
0
10
20
30
25
50
75
100
2010
2015
2020
2025
2030
PopART 2014-2030
PopART 2014-2017
No PopART
(Standard care)

S2 C

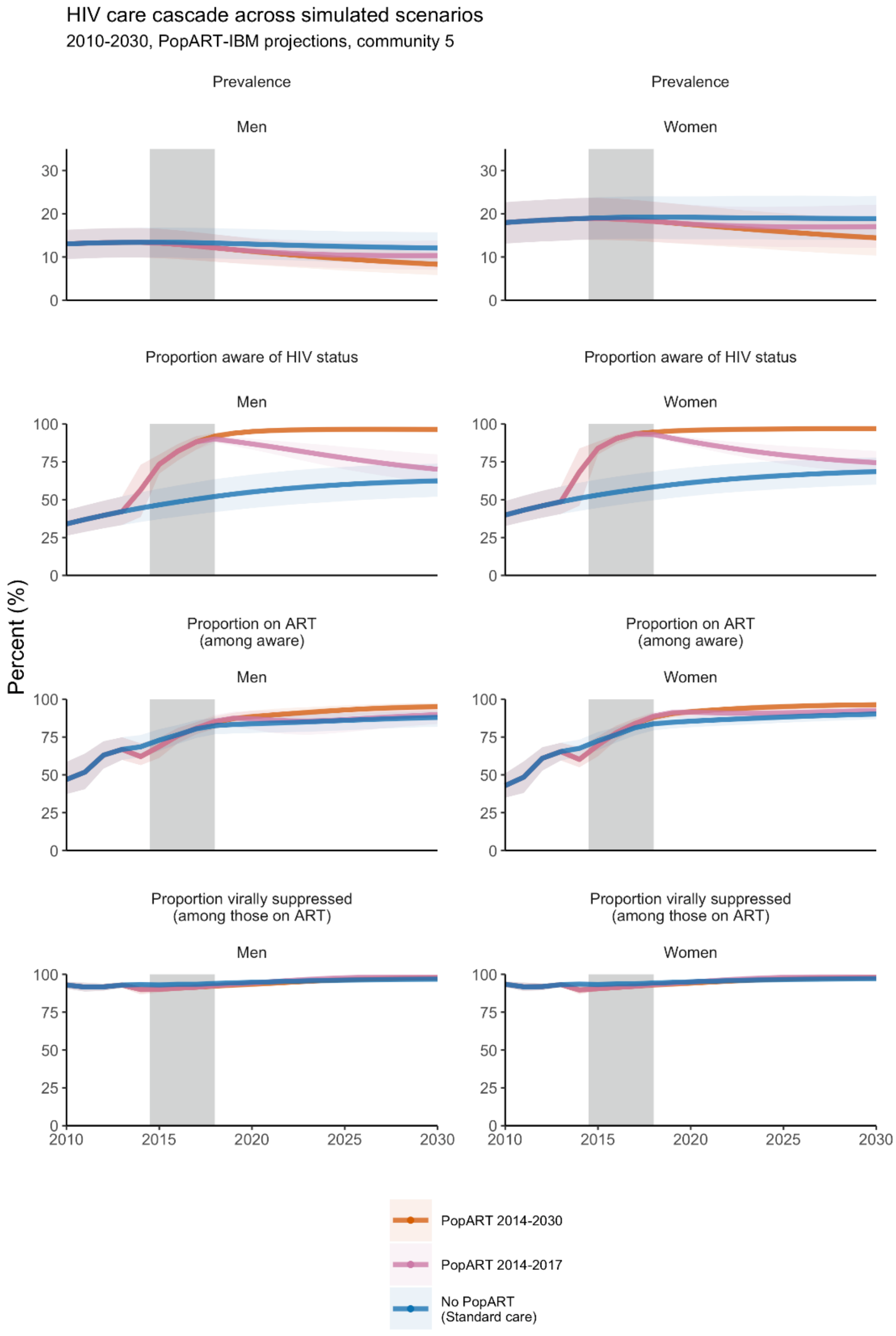
HIV care cascade across simulated scenarios
2010-2030, PopART-IBM projections, community 5
Prevalence
Men
Prevalence
Women
Proportion aware of HIV status
Men
Proportion aware of HIV status
Women
Proportion on ART
(among aware)
Men
Proportion on ART
(among aware)
Women
Proportion virally suppressed
(among those on ART)
Men
Proportion virally suppressed
(among those on ART)
Women
Percent (%)
0
10
20
30
0
25
50
75
100
2010
2015
2020
2025
2030
PopART 2014-2030
PopART 2014-2017
No PopART
(Standard care)

S2 D

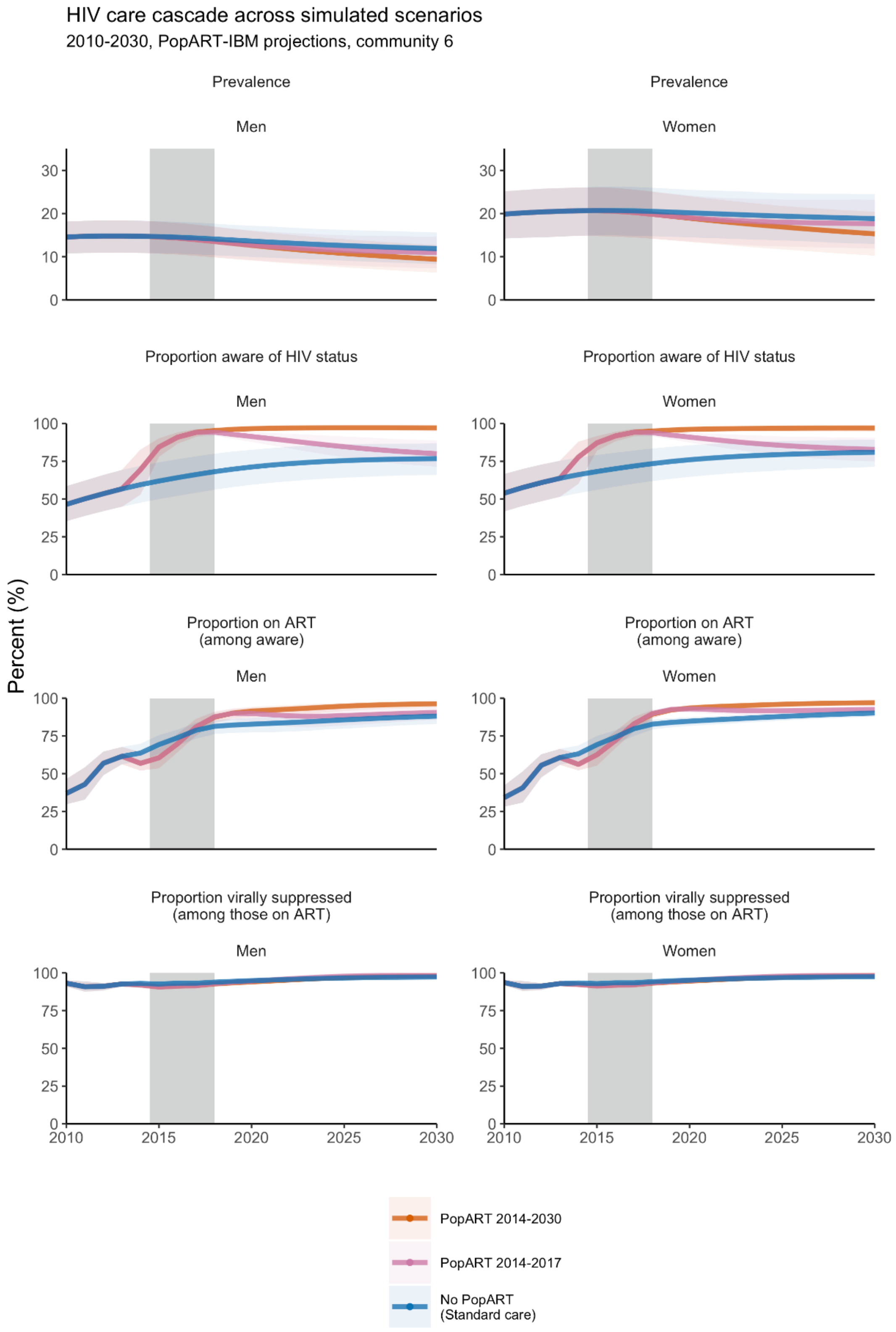
HIV care cascade across simulated scenarios
2010-2030, PopART-IBM projections, community 6
Prevalence
Men
Women
Proportion aware of HIV status
Proportion on ART
(among aware)
Proportion virally suppressed
(among those on ART)
Percent (%)
PopART 2014-2030
PopART 2014-2017
No PopART
(Standard care)

S2 E

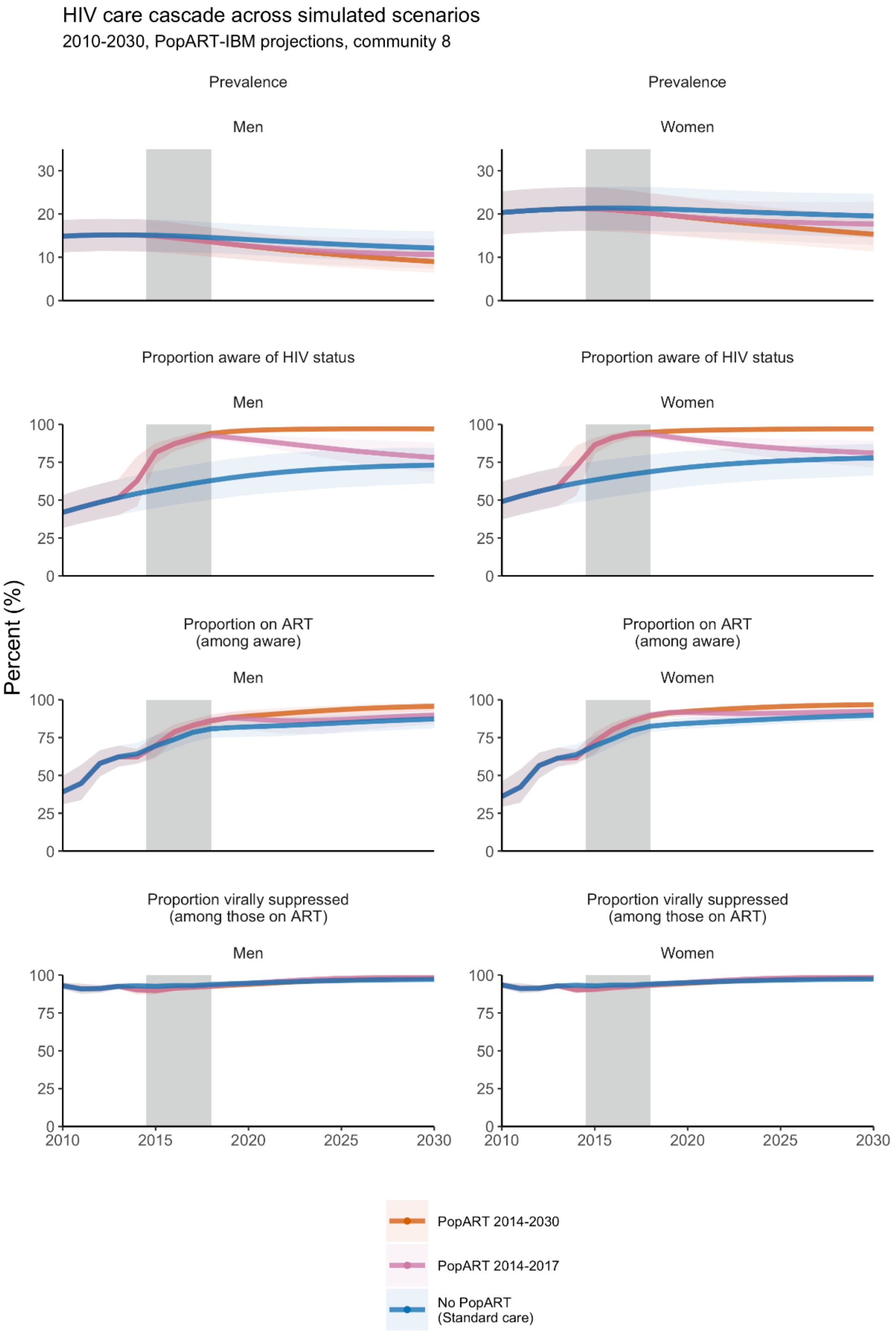
HIV care cascade across simulated scenarios
2010-2030, PopART-IBM projections, community 8
Prevalence
Men
Prevalence
Women
Proportion aware of HIV status
Men
Proportion aware of HIV status
Women
Proportion on ART
(among aware)
Men
Proportion on ART
(among aware)
Women
Proportion virally suppressed
(among those on ART)
Men
Proportion virally suppressed
(among those on ART)
Women
Percent (%)
0
10
20
30
0
25
50
75
100
2010
2015
2020
2025
2030
PopART 2014-2030
PopART 2014-2017
No PopART
(Standard care)

S2 F

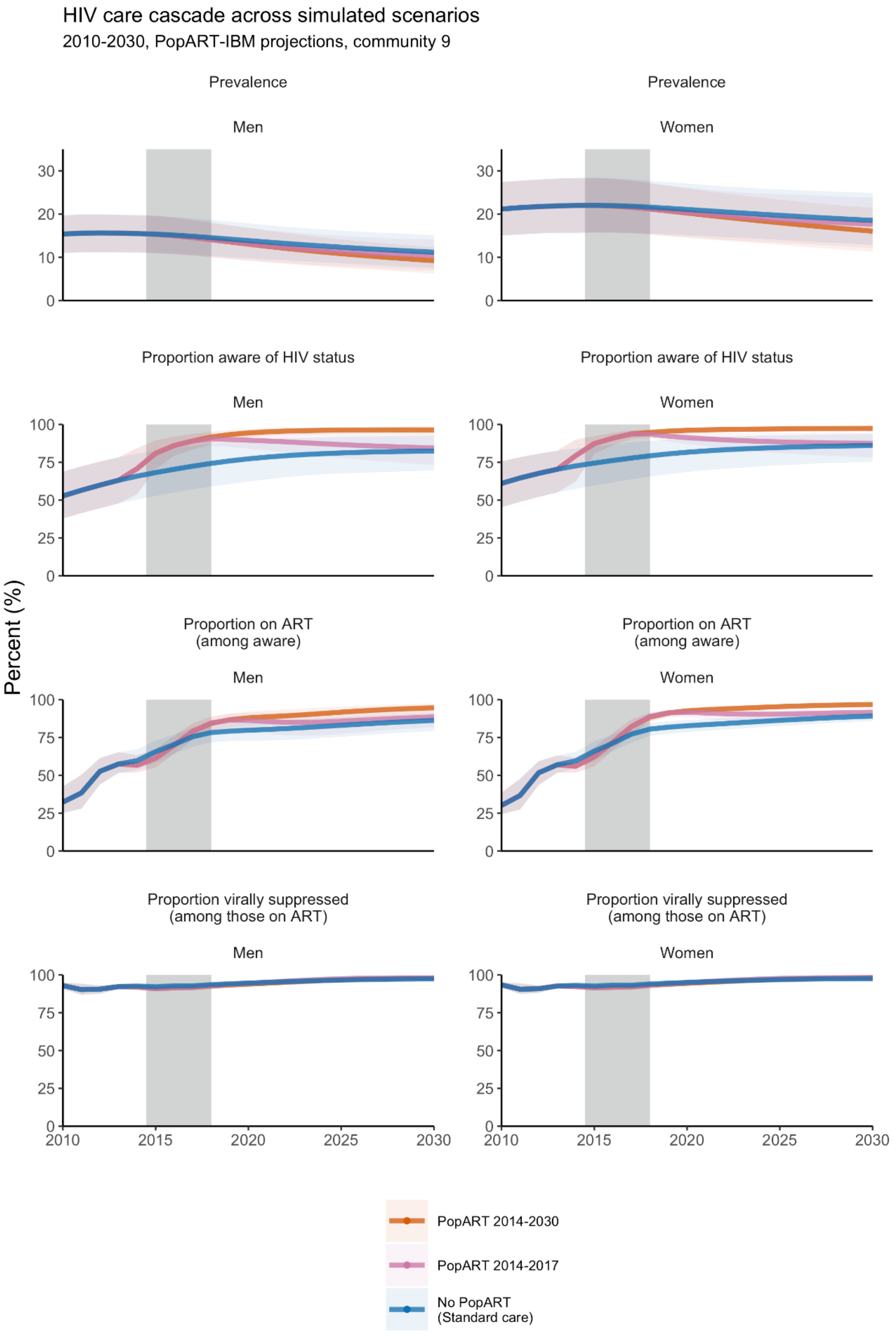

HIV care cascade across simulated scenarios
2010-2030, PopART-IBM projections, community 9
Prevalence
Men
Women
Proportion aware of HIV status
Proportion on ART
(among aware)
Proportion virally suppressed
(among those on ART)
Percent (%)
0
10
20
30
25
50
75
100
2010
2015
2020
2025
2030
PopART 2014-2030
PopART 2014-2017
No PopART
(Standard care)

S2 G

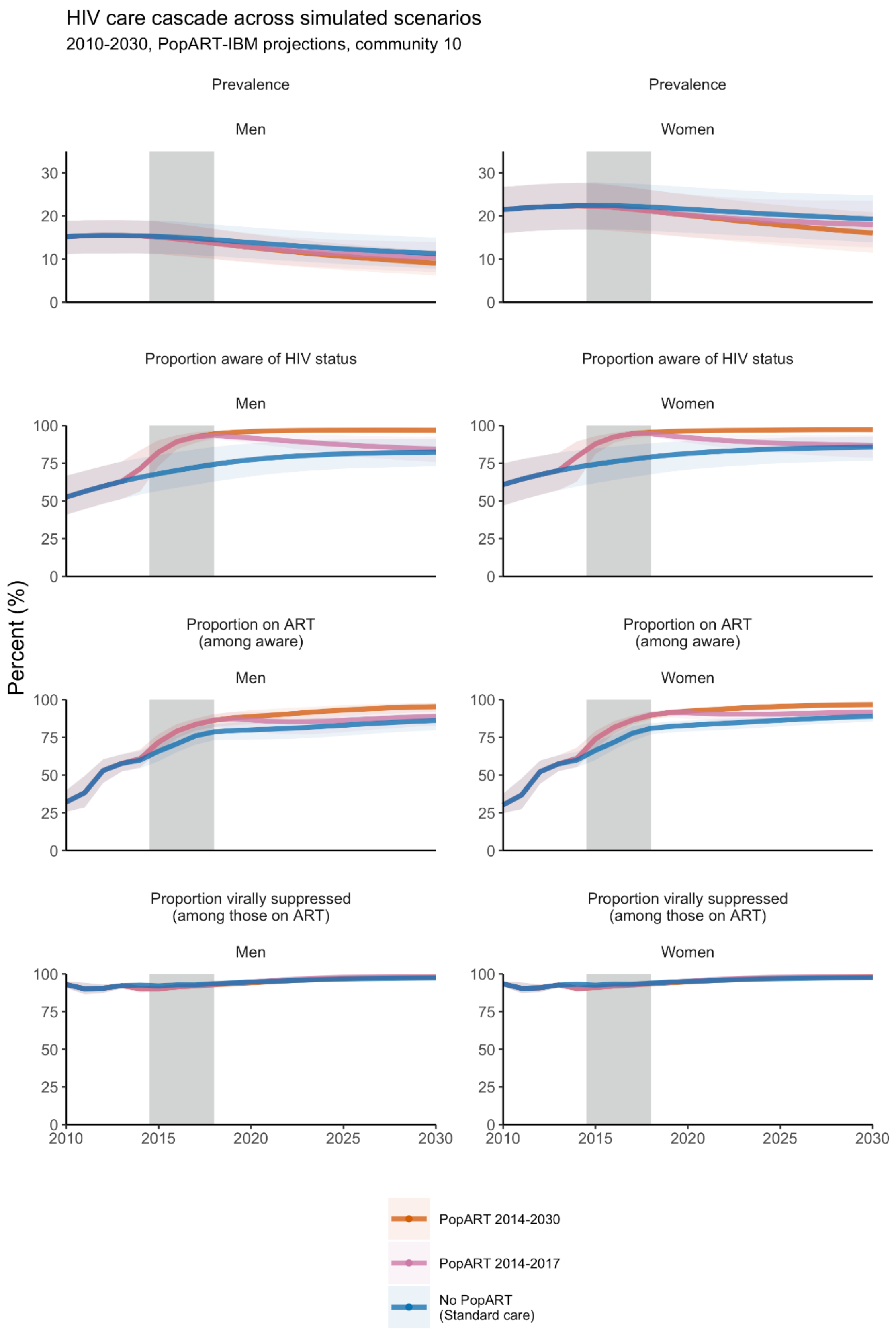
HIV care cascade across simulated scenarios
2010-2030, PopART-IBM projections, community 10
Prevalence
Men
Women
Proportion aware of HIV status
Proportion on ART
(among aware)
Proportion virally suppressed
(among those on ART)
Percent (%)
0
10
20
30
25
50
75
100
2010
2015
2020
2025
2030
PopART 2014-2030
PopART 2014-2017
No PopART
(Standard care)

S2 H

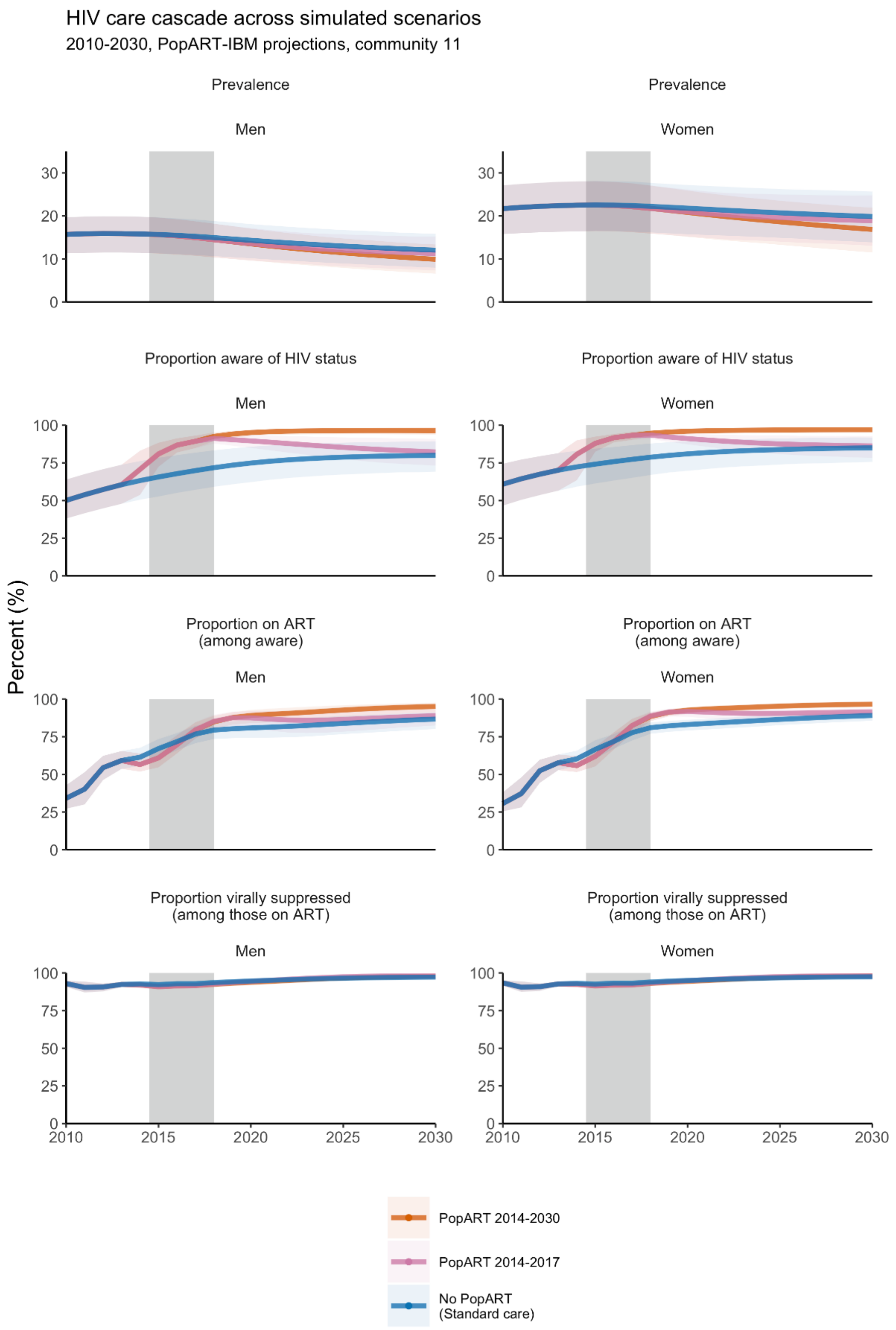
HIV care cascade across simulated scenarios
2010-2030, PopART-IBM projections, community 11
Prevalence
Men
Women
Proportion aware of HIV status
Proportion on ART
(among aware)
Proportion virally suppressed
(among those on ART)
Percent (%)
0
10
20
30
25
50
75
100
2010
2015
2020
2025
2030
PopART 2014-2030
PopART 2014-2017
No PopART
(Standard care)

S2 I

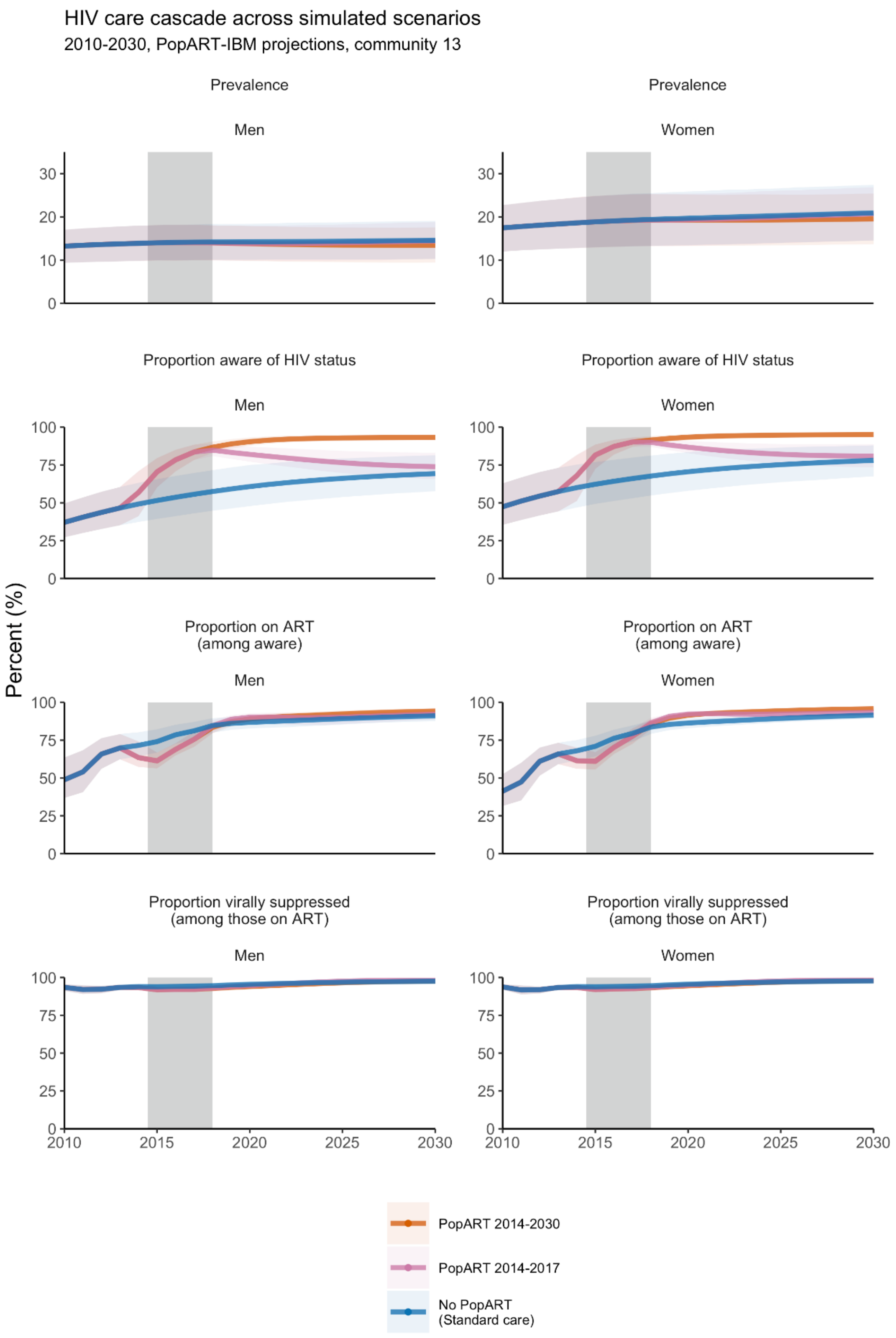

HIV care cascade across simulated scenarios
2010-2030, PopART-IBM projections, community 13
Prevalence
Men
Women
Proportion aware of HIV status
Proportion on ART (among aware)
Proportion virally suppressed (among those on ART)
Percent (%)
0
10
20
30
25
50
75
100
2010
2015
2020
2025
2030
PopART 2014-2030
PopART 2014-2017
No PopART (Standard care)

S2 J

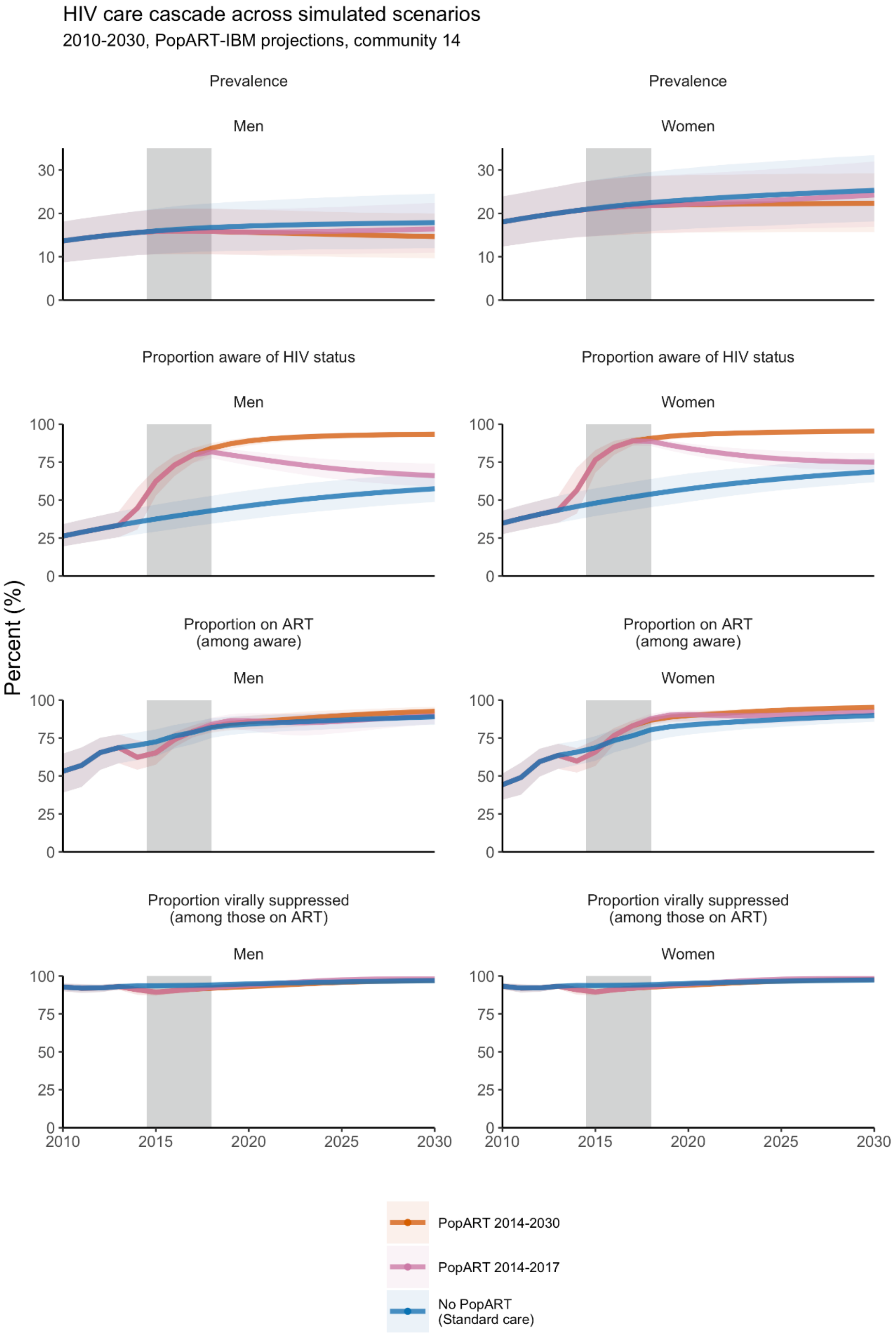
HIV care cascade across simulated scenarios
2010-2030, PopART-IBM projections, community 14
Prevalence
Men
Women
Proportion aware of HIV status
Proportion on ART
(among aware)
Proportion virally suppressed
(among those on ART)
Percent (%)
0
10
20
30
25
50
75
100
2010
2015
2020
2025
2030
PopART 2014-2030
PopART 2014-2017
No PopART
(Standard care)

S2 K

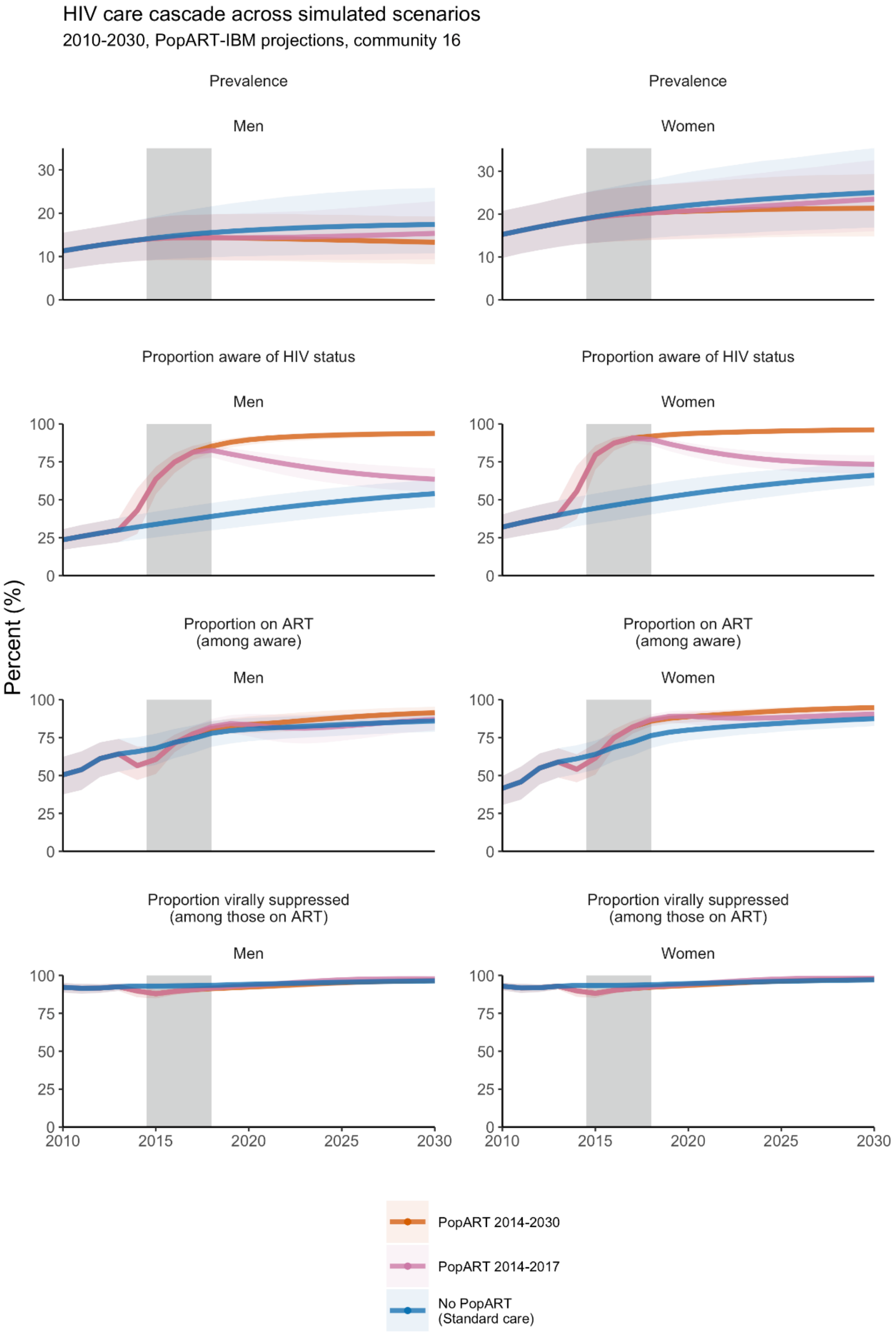
HIV care cascade across simulated scenarios
2010-2030, PopART-IBM projections, community 16
Prevalence
Men
Women
Proportion aware of HIV status
Proportion on ART
(among aware)
Proportion virally suppressed
(among those on ART)
Percent (%)
0
10
20
30
25
50
75
100
2010
2015
2020
2025
2030
PopART 2014-2030
PopART 2014-2017
No PopART
(Standard care)

S2 L

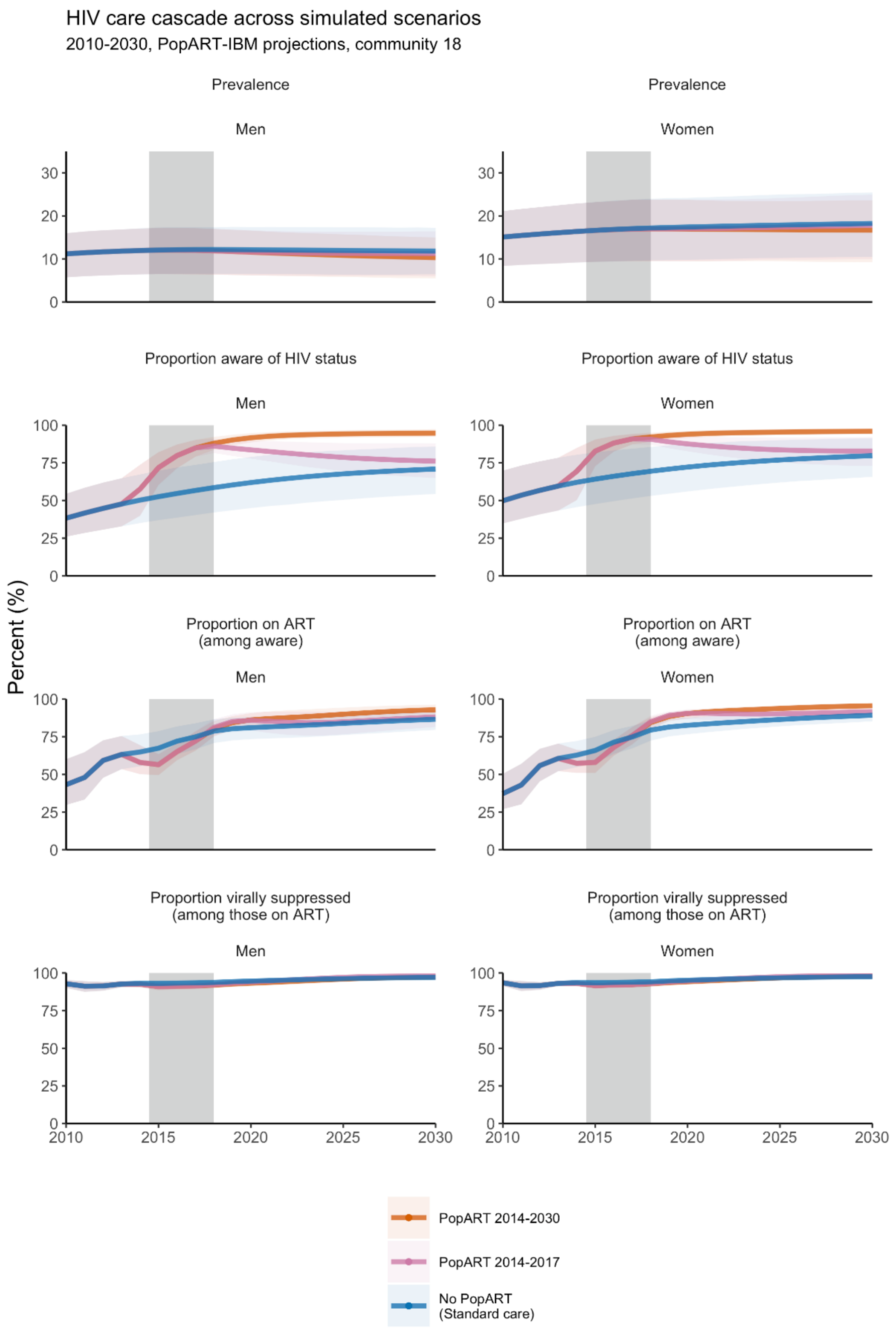
HIV care cascade across simulated scenarios
2010-2030, PopART-IBM projections, community 18
Prevalence
Men
Prevalence
Women
Proportion aware of HIV status
Men
Proportion aware of HIV status
Women
Proportion on ART
(among aware)
Men
Proportion on ART
(among aware)
Women
Proportion virally suppressed
(among those on ART)
Men
Proportion virally suppressed
(among those on ART)
Women
Percent (%)
0
10
20
30
0
25
50
75
100
2010
2015
2020
2025
2030
PopART 2014-2030
PopART 2014-2017
No PopART
(Standard care)

S2 M

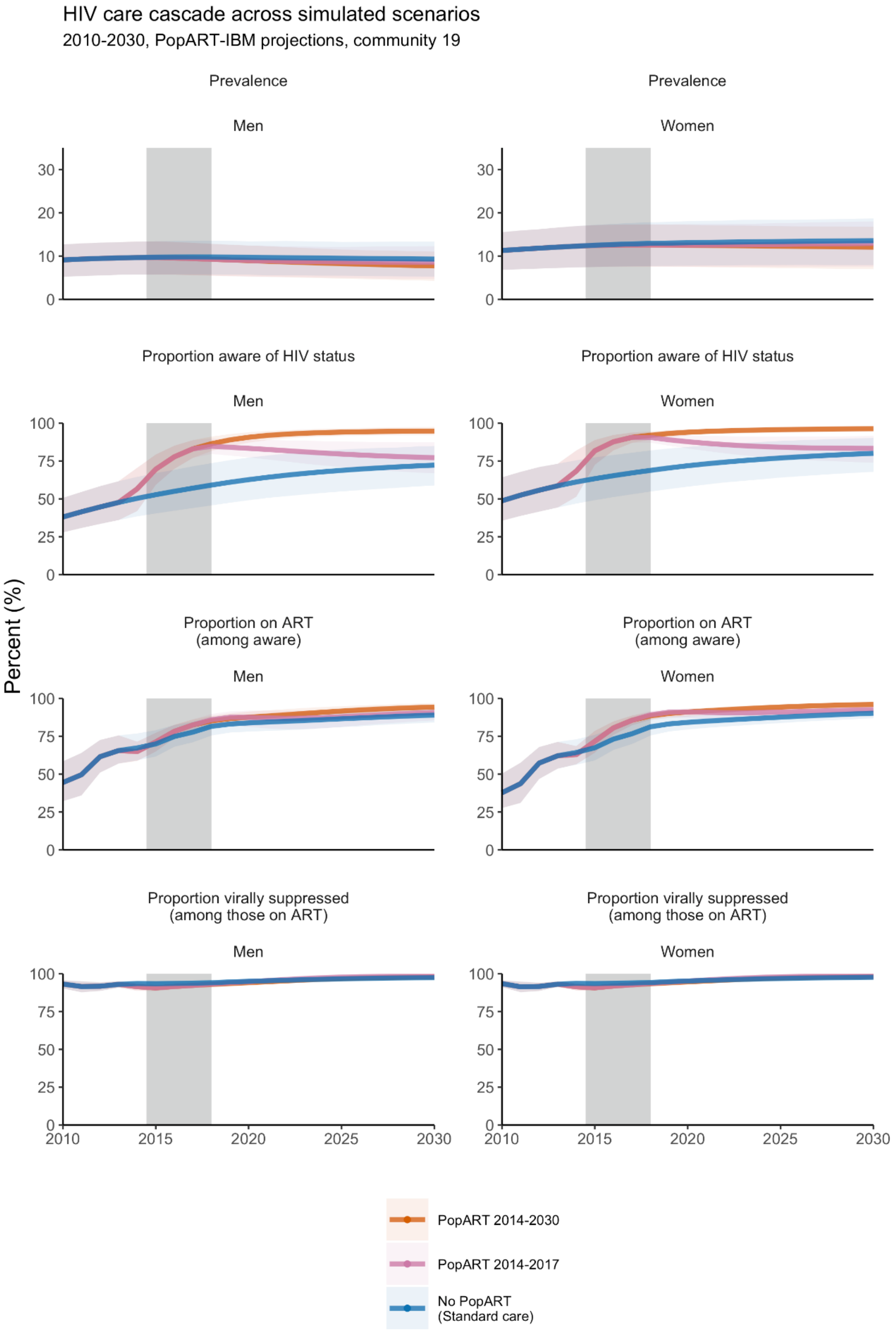
HIV care cascade across simulated scenarios
2010-2030, PopART-IBM projections, community 19
Prevalence
Men
Prevalence
Women
Proportion aware of HIV status
Men
Proportion aware of HIV status
Women
Proportion on ART
(among aware)
Men
Proportion on ART
(among aware)
Women
Proportion virally suppressed
(among those on ART)
Men
Proportion virally suppressed
(among those on ART)
Women
Percent (%)
0
10
20
30
25
50
75
100
2010
2015
2020
2025
2030
PopART 2014-2030
PopART 2014-2017
No PopART
(Standard care)

S2 N

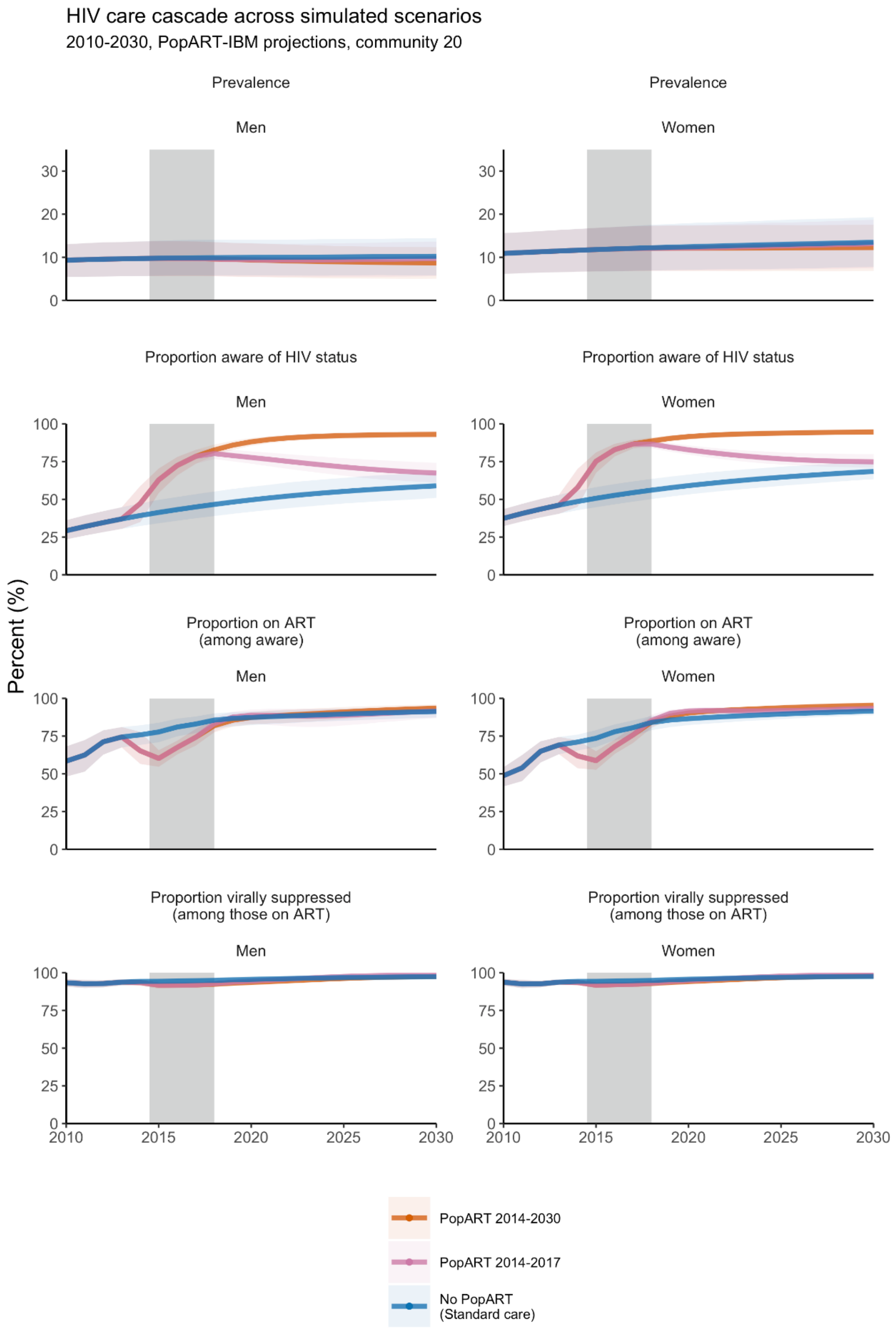

Figure S2 A-N: HIV care cascades by PopART trial community

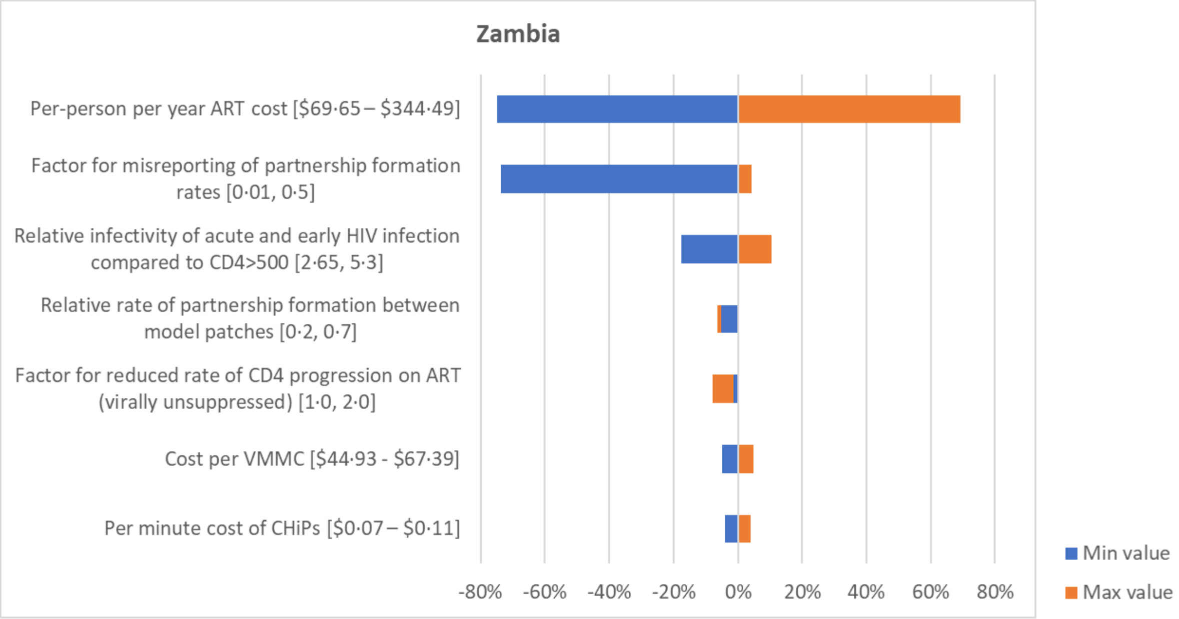

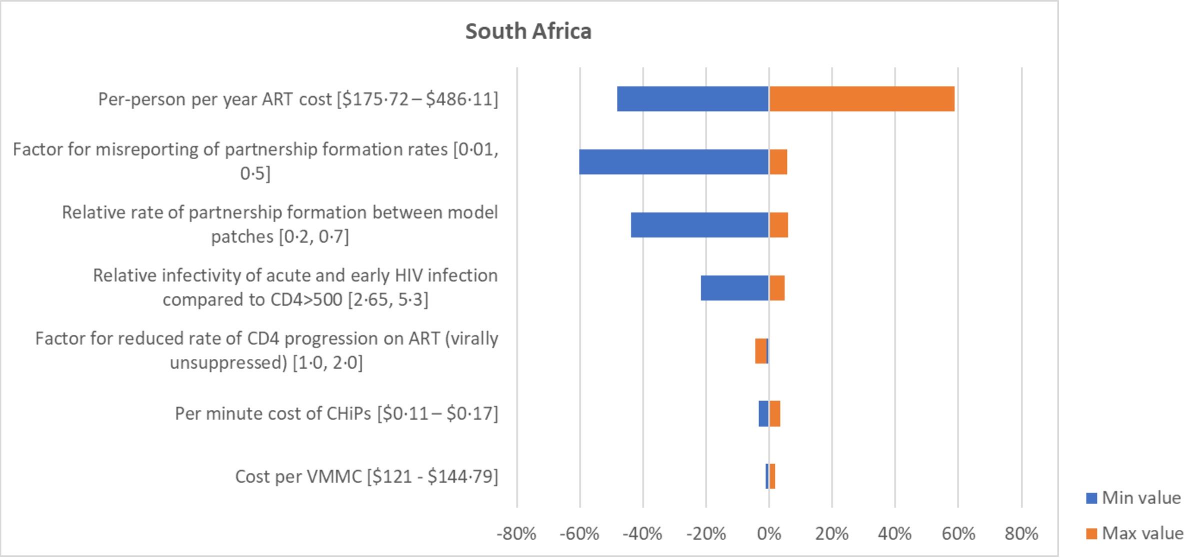

Figure S3: One-way Parameter Sensitivity Analysis

Notes for figure S1: 0% represents base case, the ICER generated under the "best fitting" parameter set with costs at mean values from Table 2 in the main text. Bars indicate the extent to which the ranges used in the uncertainty analysis influence the ICER as % change from the base case.

Table S1:Disability weights

	Disability weights
Uninfected	0
HIV-positive, CD4 count >350 cells per µl (untreated)	0·053
HIV-positive, CD4 count >200-350 cells per µl (untreated)	0·221
HIV-positive, CD4 count <=200 cells per µl (untreated)	0·547
HIV-positive, on antiretroviral therapy	0·053
Deceased	1

Note: Disability weights taken from Salomon, Vos [13]

Table S2: CHiPs Time Spent Per Person

CHiPs time per person	Country	Average time per person in minutes
Administration and travel	Zambia	36
	South Africa	22
HIV-positive test result	Zambia	112
	South Africa	85
HIV-negative test result	Zambia	70
	South Africa	52

Table S3: Cost-per-person-per year on ART (US$)

**Zambia**		**South Africa**	
Community 1	$344·50	Community 13	$252·73
Commuity 2	$336·75	Community 14	$300·43
Community 3	$149·94	Community 15	$211·45
Community 4	$191·05	Community 16	$348·26
Community 5	$251·59	Community 17	$320·85

Community 6	$190·17	Community 18	$175·72
Community 7	$103·51	Community 19	$427·60
Community 8	$69·65	Community 20	$486·12
Community 9	$98·60	Community 21	$2040·06
Community 10	$268·60		
Community 11	$339·15		
Community 12	$206·43		

Table S4: ICERs at discount rates of 1% and 8%

	**PopART 2014-30 (uncertainty interval)**			**PopART 2014-17 (uncertainty interval)**		
	**3%**	**1%**	**8%**	**3%**	**1%**	**8%**
**Zambia**						
Infections averted	$2,111 ($1,827 - $2,462)	$2,061 ($1,781 - $2,401)	$2,247 ($1,953 - $2,620)	$1,318 ($1,098 - $1,591)	$1,208 ($996 - $1,473)	$1,590 ($1,346 - $1,890)
DALYs averted	$593 ($526 - $674)	$554 ($491-$631)	$707 ($628-$803)	$258 ($225 - $298)	$218 ($189-$252)	$382 ($335-$438)
**South Africa**						
Infections averted	$3,248 ($2,472 - $3,963)	$3,169 ($2,406 - $3,864)	$3,474 ($2,664 - $4,266)	$2,236 ($1,601-$2,916)	$2,076 ($1,465 - $2,735)	$2,650 ($1,920 - $3,864)
DALYs averted	$645 ($538 - $757)	$599 ($499-$705)	$784 ($657-$915)	$326 ($266-$391)	$278 ($226-$336)	$472 ($393-$561)

Notes for Table S3: ICERS are median and 95% credible intervals at discount rates of 1% and 8%. ICERs at 3% are presented for reference.

Table S5: ICERs by model time horizons

	PopART 2014-30 (uncertainty interval)			PopART 2014-17 (uncertainty interval)		
	**2030**	**2035**	**2040**	**2030**	**2035**	**2040**
**Zambia**						
Infections averted	$2,111 ($1,827 - $2,462)	$2,007 ($1,734- $2,351)	$1,941 ($1,662- $2,283)	$1,318 ($1,098- $1,591)	$1,229 ($1,004- $1,507)	$1,185 ($946 - $1,471)
DALYs averted	$593 ($526 - $674)	$448 ($396- $514)	$367 ($323- $426)	$258 ($225 - $298)	$171 ($149- $199)	$129 ($111 - $150)
**South Africa**						
Infections averted	$3,248 ($2,472 - $3,963)	$3,135 ($2,375- $3,838)	$3,082 ($2,381- $3,968)	$2,236 ($1,601- $2,916)	$2,148 ($1,504- $2,870)	$2,124 ($1,490- $2,855)
DALYs averted	$645 ($538 - $757)	$473 ($393- $560)	$379 ($313- $447)	$326 ($266-$391)	$223 ($179- $270)	$173 ($137 - $211)

Notes for Table S4: ICERS are median and 95% credible intervals. ICERs for 2030 are presented for reference.

Table S6: Results by trial arm

**PopART 2014-30 Scenario**	**Main results (Arms A and B)**	**ICER Arm A communities only**	**ICER Arm B communities only**
Zambia	$593 ($526-$674)	$540 ($465-$649)	$658 ($558-$792)
South Africa	$645 ($538-$757)	$536 ($421-$681)	$811 ($655-$1,027)

Notes for Table S5: ICERS are median and 95% credible intervals.

Table S7: Parameter ranges for one-way sensitivity analysis

**Parameter**	**Range (min-max) – Zambia**	**Range (min-max) – South Africa**
Per-person-per year cost of ART	[$69·65 – $344·49]	[$175·72 – $486·11]
Cost per VMMC	[$44·93 - $67·39]	[$121 - $144·79]
Per minute cost of CHiPs	[$0·07 – $0·11]	[$0·11 – $0·17]
Factor for misreporting of partnership formation rates	[0·01, 0·5]	

Relative infectivity of acute and early HIV infection compared to CD4 > 500	[2·65, 5·3]
Factor for reduced rate of CD4 progression on ART (virally unsuppressed)	[1·0, 2·0]
Relative rate of partnership formation between model patches	[0·2, 0·7]

References

1. Weiss HA, Quigley MA, Hayes RJ. Male circumcision and risk of HIV infection in sub-Saharan Africa: a systematic review and meta-analysis. *AIDS* 2000; **14**(15): 2361-70.
2. Garenne M. Long-term population effect of male circumcision in generalised HIV epidemics in sub-Saharan Africa. *Afr J AIDS Res* 2008; **7**(1): 1-8.
3. Bailey RC, Moses S, Parker CB, et al. Male circumcision for HIV prevention in young men in Kisumu, Kenya: a randomised controlled trial. *Lancet* 2007; **369**(9562): 643-56.
4. Gray RH, Kigozi G, Serwadda D, et al. Male circumcision for HIV prevention in men in Rakai, Uganda: a randomised trial. *Lancet* 2007; **369**(9562): 657-66.
5. Pickles M, Cori A, Floyd S, et al. Exploring the Effectiveness of Traditional Circumcision Practices in Preventing HIV. CROI. Boston, Massachusetts; 2016.
6. Fraser C, Hollingsworth TD, Chapman R, de Wolf F, Hanage WP. Variation in HIV-1 set-point viral load: epidemiological analysis and an evolutionary hypothesis. *Proc Natl Acad Sci U S A* 2007; **104**(44): 17441-6.
7. Cori A, Pickles M, van Sighem A, et al. CD4+ cell dynamics in untreated HIV-1 infection: overall rates, and effects of age, viral load, sex and calendar time. *AIDS* 2015; **29**(18): 2435-46.
8. Beaumont MA, Cornuet J-M, Marin J-M, Robert CP. Adaptive approximate Bayesian computation. *Biometrika* 2009; **96**(4): 983-90.
9. Lenormand M, Jabot F, Deffuant G. Adaptive approximate Bayesian computation for complex models. *Computational Statistics* 2013; **28**(6): 2777-96.
10. ICF. Demographic and Health Surveys (various). Rockville, Maryland: ICF.
11. WHO. Tables of Costs and Prices used in WHO-CHOICE Analysis. World Health Organization; 2018.
12. CHAI. HIV Market Report: The state of the HIV treatment, testing, and prevention markets in low- and middle-income countries, 2017-20222018. https://clintonhealthaccess.org/content/uploads/2018/09/2018-HIV-Market-Report_FINAL.pdf (accessed.
13. Salomon JA, Vos T, Hogan DR, et al. Common values in assessing health outcomes from disease and injury: disability weights measurement study for the Global Burden of Disease Study 2010. *The Lancet* 2012; **380**(9859): 2129-43.
