# Supplementary material for: Cost and cost-effectiveness of a universal HIV testing and treatment intervention in Zambia and South Africa: evidence and projections from the HPTN 071 (PopART) trial
Source: Lancet Glob Health. 2021 Mar 12;9(5):e668–80. doi: 10.1016/S2214-109X(21)00034-6 (PMC8050197; doi:10.1016/S2214-109X(21)00034-6)
Supplement: Supplementary appendix 2 [file mmc2.pdf]

THE LANCET
Global Health

Supplementary appendix 2

This appendix formed part of the original submission and has been peer reviewed. We post it as supplied by the authors.

Supplement to: Thomas R, Probert WJM, Sauter R, et al. Cost and cost-effectiveness of a universal HIV testing and treatment intervention in Zambia and South Africa: evidence and projections from the HPTN 071 (PopART) trial. *Lancet Glob Health* 2021; published online March 12. https://doi.org/10.1016/S2214-109X(21)00034-6.

HPTN 071 (PopART) Data Sharing policy

Background

NIAID, HPTN, LSHTM, and other PopART partners have general expectations and/or policies to make research data available to other researchers in a collaborative manner. However, there is need for an agreed, comprehensive and detailed data sharing policy specific to this study to be widely shared so that all stakeholders share the same expectations for when and what data will be made available, and through what mechanisms. Such a policy will ensure that HPTN 071 protocol team members and stakeholders have a period of priority access to the data, while still providing a pathway for more public access over time, and will strive to ensure that public use represents the data responsibly. As well, a comprehensive policy will avoid reliance on multiple "one-off" data sharing policies or mechanisms that might otherwise arise and be inconsistent with one another. It will enable the team to address expectations and requirements of funding agencies, journals, conferences and the broader scientific community, and will maximise the public policy and health benefit that may be derived from the data.

Types of HPTN 071 (PopART) Data Addressed by this Policy

There are two main types of HPTN071 study data:

1. Data obtained from the population cohort (PC)
   a. Survey data collected by PC field teams
   b. Data obtained from analysis of blood samples obtained from PC participants during household visits by the PC field teams
2. Data obtained by intervention field teams
   a. Data obtained during household visits in Arm A & B communities. Intervention data available aggregated by sex, age group and community.

Principles forming the basis of the HPTN 071 (PopART) data sharing policy

- The HPTN 071 (PopART) protocol team agrees that wider access to research data is in the public interest.

- Secondary analyses arising as a result of data sharing should meet the same high standards expected of the HPTN and HPTN 071 (PopART) analyses.

- The HPTN is committed to building scientific capacity among early career investigators at the research sites in Africa, and this requires that the study team should have exclusive access to the data for a defined period.

- Data arising from research with human subjects have special ethical considerations in terms of confidentiality and consent.

- Because the HPTN 071 (PopART) study is an interventional trial designed to answer a specific set of research questions, the data may not always be appropriate to answer other questions.

- Conclusions derived from misunderstandings or erroneous analyses of data can harm the reputation of a study, destroy the trust of the community, and discourage further participation in research. Therefore, the HPTN 071 (PopART) protocol team has a responsibility to ensure that data are accessed only by legitimate investigators who have agreed to abide by the provisions of this policy.

Key aspects of the HPTN 071 (PopART) data sharing policy

- Researchers from the HPTN 071 (PopART) study team who collected data have a legitimate interest in benefiting from their investment of time and effort, as well as a commitment to supporting capacity building for early career investigators at the study sites. Therefore, the study team will have a period of *exclusive use* before the data are made available for sharing.

    1. Exclusive use will be for a fixed period of 1 year after the publication of the primary results in the New England Journal of Medicine on 18 July 2019.
    2. De-identified analysis datasets for the primary publication will be released to 3ie following publication of the primary manuscript, for replication purposes. Analysis datasets supporting other manuscripts will be posted as required by journals at the time of publication.
    3. Data may be made available to researchers external to the study team earlier, with specific permission of the Protocol Chairs and Protocol Statistician when this does not conflict with the publication plans for the study.

- During and after the period of exclusive protocol team use, protocol team members with approved publication concepts are provided access to HPTN 071 PopART data stored at the

HPTN SDMC by submission of a signed HPTN 071 Data Access Agreement. Protocol team members agree that they will only use the data for the analyses in the approved publication concept.

- During and after the period of exclusive protocol team use, protocol team members may use the data to pursue analyses agreed upon by protocol team leadership but not explicitly described in an approved publication concept, especially those requested by study funders or policymakers.

- After the period of exclusive use, data will be made available to users outside of the protocol team after an application and approval process (controlled public access).

- Requests for data are made in writing to the HPTN 071 (PopART) Publications Working Group, using a standard Data Access Proposal Form. Proposals are reviewed by the Publications Working Group, and access to study data is facilitated by the HPTN Statistical and Data Management Center (SDMC).

- Researchers external to the study protocol team who are granted access to data are encouraged to engage with the HPTN 071 (PopART) study team to ensure they have sufficient understanding of the study and the data elements.

- Any publications arising from the shared data must acknowledge the investigators who collected the data, the institutions involved, and the funding sources. A standard acknowledgement statement will be provided.

Storage for data sharing

- Data will be stored securely at the HPTN SDMC according to HPTN and HPTN 071 (PopART) Standard Operating Procedures (SOPs) and access provided to approved applicants.

- Authorized users will be granted access to all study data relevant to addressing the research question posed.

Standards for data sharing

Data cannot be used effectively unless they are thoroughly documented and their collection methods

understood. Therefore, in general, data are made available for sharing when they are cleaned, documented and the appropriate metadata are in place. Documentation includes detailed descriptions of how the data were collected and coded, and how lab results were obtained. The annotated questionnaire will also be included. Data available for sharing will be from the final ‘locked’ database.

The following standards will be observed:

- Data will be de-identified before release for sharing, with all direct personal identifiers removed. Where there are indirect identifiers that could lead to deductive disclosure (e.g. GPS coordinates for a person’s house), these will be modified or removed from the dataset. Any requests for access to a dataset that includes identifiers will need to be negotiated with the HPTN 071 (PopART) Publications Working Group. If necessary, such requests may need to be referred to relevant research and ethics committees.
- Data may be in separate files, but each file will have a data dictionary that identifies the key fields needed to merge files
- Data documentation will also include the names and institutions of the PIs who collected the data and details of the funding source of the study

Conditions of access for non-protocol team members

All requests to share data must be made in writing using the HPTN 071 (PopART) Data Access Application Form. Where relevant, an accompanying proposal detailing the study questions and analysis methods should be submitted as an appendix to the application form. Applications will be reviewed by the HPTN 071 Publications Working Group.

In order for access to be considered, the applicant must meet the following requirements:

- provide evidence of being a bona fide researcher in a field relevant to the study or on associated statistical methods (e.g. relevant peer-reviewed research that can be found on PubMed, or a successful application to a relevant, independent data access committee). If the primary investigator who will be using the data does not meet these criteria, they must identify a mentor who will take responsibility for supervising them and guaranteeing their compliance with this policy
- have an experienced statistician on their research team
- be willing to sign a data access agreement

- ☐ agree to notify the HPTN SDMC if any errors are identified in the data
- ☐ agree not to share the data with third parties without permission from the HPTN 071 Publications Working Group
- ☐ agree to share the methods and results of all the analyses using HPTN 071 (PopART) data with the HPTN 071 protocol chairs and protocol statistician when completed, and certainly no later than when analyses have been accepted for publication or presentation.

Priority will be given to applicants whose proposals include a plan for sharing analysis skills with site researchers. Access decisions will take into consideration the planned publications by the HPTN 071 (PopART) protocol team. The committee will make its decision within 4 weeks of the application being received. The aim will be that the data will then be transferred within 4 weeks after a positive decision. Where this is not possible, the Protocol Statistician at the HPTN SDMC will inform the HPTN 071 (PopART) Publications Working Group and the applicant in writing and will negotiate a later date.

Any publications arising from the shared data must acknowledge the research team who collected the data, the institutions involved, and funding sources, and must state that HPTN 071 (PopART) investigators were not involved in the analysis (except in the case where there are protocol team collaborators). This acknowledgement statement will be provided to investigators with whom HPTN 071 (PopART) data are shared.
